# *Candida* and the Gram-positive trio: testing the vibe in the ICU patient microbiome using structural equation modelling of literature derived data

**DOI:** 10.1186/s12982-022-00116-9

**Published:** 2022-08-18

**Authors:** James C. Hurley

**Affiliations:** 1 Department of Rural Health, Melbourne Medical School, University of Melbourne, Melbourne, VIC Australia; 2 Division of Internal Medicine, Ballarat Health Services, Ballarat, VIC Australia

**Keywords:** Bacteremia, *Staphylococcus aureus*, Antibiotic prophylaxis, Study design, Intensive care, Mechanical ventilation, Selective digestive decontamination, Generalized structural equation model

## Abstract

**Background:**

Whether *Candida* interacts with Gram-positive bacteria, such as *Staphylococcus aureus,* coagulase negative Staphylococci (CNS) and *Enterococci,* to enhance their invasive potential from the microbiome of ICU patients remains unclear. Several effective anti-septic, antibiotic, anti-fungal, and non-decontamination based interventions studied for prevention of ventilator associated pneumonia (VAP) and other ICU acquired infections among patients receiving prolonged mechanical ventilation (MV) are known to variably impact Candida colonization. The collective observations within control and intervention groups from numerous ICU infection prevention studies enables tests of these postulated microbial interactions in the clinical context.

**Methods:**

Four candidate generalized structural equation models (GSEM), each with *Staphylococcus aureus, CNS* and *Enterococci* colonization, defined as latent variables, were confronted with blood culture and respiratory tract isolate data derived from 460 groups of ICU patients receiving prolonged MV from 283 infection prevention studies.

**Results:**

Introducing interaction terms between *Candida* colonization and each of *S aureus* (coefficient + 0.40; 95% confidence interval + 0.24 to + 0.55), CNS (+ 0.68; + 0.34 to + 1.0) and *Enterococcal* (+ 0.56; + 0.33 to + 0.79) colonization (all as latent variables) improved the fit for each model. The magnitude and significance level of the interaction terms were similar to the positive associations between exposure to topical antibiotic prophylaxis (TAP) on *Enterococcal* (+ 0.51; + 0.12 to + 0.89) and Candida colonization (+ 0.98; + 0.35 to + 1.61) versus the negative association of TAP with *S aureus* (− 0.45; − 0.70 to − 0.20) colonization and the negative association of anti-fungal exposure and Candida colonization (− 1.41; − 1.6 to − 0.72).

**Conclusions:**

GSEM modelling of published ICU infection prevention data enables the postulated interactions between *Candida* and Gram-positive bacteria to be tested using clinically derived data. The optimal model implies interactions occurring in the human microbiome facilitating bacterial invasion and infection. This interaction might also account for the paradoxically high bacteremia incidences among studies of TAP in ICU patients.

**Supplementary Information:**

The online version contains supplementary material available at 10.1186/s12982-022-00116-9.

## Keypoints

- GSEM modelling of published ICU infection prevention data from > 250 studies enables a test of and provides support to the interaction between Candida and Gram-positive bacteria.
- The various ICU infection prevention interventions may each broadly impact the patient microbiome.

**Supplementary Information:**

The online version contains supplementary material available at 10.1186/s12982-022-00116-9.

## Introduction

While *Candida* rarely causes ventilator associated pneumonia (VAP), and blood stream infections (BSI) with Candida (candidemia) are uncommon in the ICU, surprisingly, *Candida* colonization is associated with higher mortality and poor patient outcomes among ICU patients receiving mechanical ventilation (MV) [1, 2]. The basis for this association remains unclear and interactions between *Candida* and bacterial colonizations causing invasive infection have been implicated from preclinical studies [3–8]. Moreover, Gram-positive bacteria, such as *Staphylococcus aureus* account for the majority of candidemia associated mixed blood stream infections among ICU patients [9] and bacterial colonization is a key determinant [10].

Evaluating the possible clinical relevance of microbial interactions is unlikely to be achieved within the constraints of a single center study. Moreover, quantifying the impact of the various interventions on not only the presence but also the biological activity of microbial colonization within the microbiome is not simple. Structural equation modelling of literature derived data offers a novel approach [11–15].

Several anti-septic, antibiotic, anti-fungal, or non-decontamination based interventions have been studied for the prevention of ICU acquired infections. These methods target bacterial and *Candida* colonization with variable specificity [12, 16]. Of note, Topical antibiotic prophylaxis (TAP) based methods appear to be the most effective but these are always used in combination regimens together with an antifungal (termed selective digestive decontamination; SDD) due to their broad microbiome effects [12, 16]. Yet surprisingly, the incidences of candidemia, VAP and bacteremia with *Staphylococcus aureus,* coagulase negative Staphylococci (CNS) and *Enterococci* are unusually high among studies of methods using TAP and moreso among the concurrent control groups of these studies [17–21]. These paradoxically high incidences are unexplained.

**Fig. 1:**
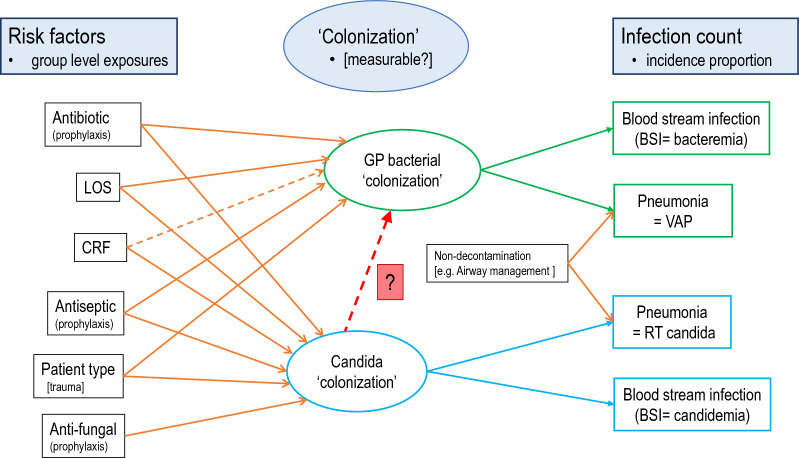
Theoretical model of established and potential factors (structural components; boxes on the left) bearing on the interaction between *bacterial* and candida colonization towards causing blood stream (BSI) and pneumonia (VAP) infections (measurement components; counts on the right). These elements are required to address the central research question (depicted by the red vertical dotted arrow labelled ‘?’) being whether the candida colonization and bacterial colonization interact to enhance invasive bacterial infections. As the colonizing candida and bacteria might change either in numbers or in activity, these are latent variables determined by the measurement components. Most individual elements are accepted although whether CRF are risk factors for bacterial colonization is unknown (sloping dotted arrow) and require testing. *CRF* candidemia risk factors, *LOS* length of ICU stay, *RT candida* respiratory tract candida (numbers of patients with VAP where Candida is identified), *VAP* ventilator associated pneumonia

The objective here is to develop candidate generalized structural equation models (GSEM) of infections arising from colonization with Candida and Gram-positive bacteria with versus without the interaction terms as postulated in the literature (Fig. 1). The optimal model emerges after confronting these models using group level infection data from published studies of ICU patient groups with various group level exposures.

## Materials and methods

Being an analysis of published work, ethics committee review of this study was not required.

### Study selection and decant of groups

The literature search uses systematic reviews of several infection prevention interventions found in The Cochrane database of systematic reviews as a starting point. These systematic reviews were identified by searching the Cochrane systematic review database using the following search terms; mechanical ventilation, ventilator associated pneumonia, blood stream infections and Candida to identify relevant systematic reviews of infection prevention interventions [22–36]. The basic inclusion criterion for individual studies identified within these systematic reviews was patient groups requiring prolonged (> 24 h) ICU stay within studies of ICU infection prevention interventions applicable to patients receiving mechanical ventilation (MV) with, as an additional inclusion criterion, group level *Candida, Staphylococcus aureus,* CNS and *Enterococcal* infection data reported. The intervention studies were classified into four categories based on the principal component of the intervention. Studies meeting these criteria but without ICU infection prevention interventions (observational studies) were sourced to provide benchmark incidence data.

**Fig. 2:**
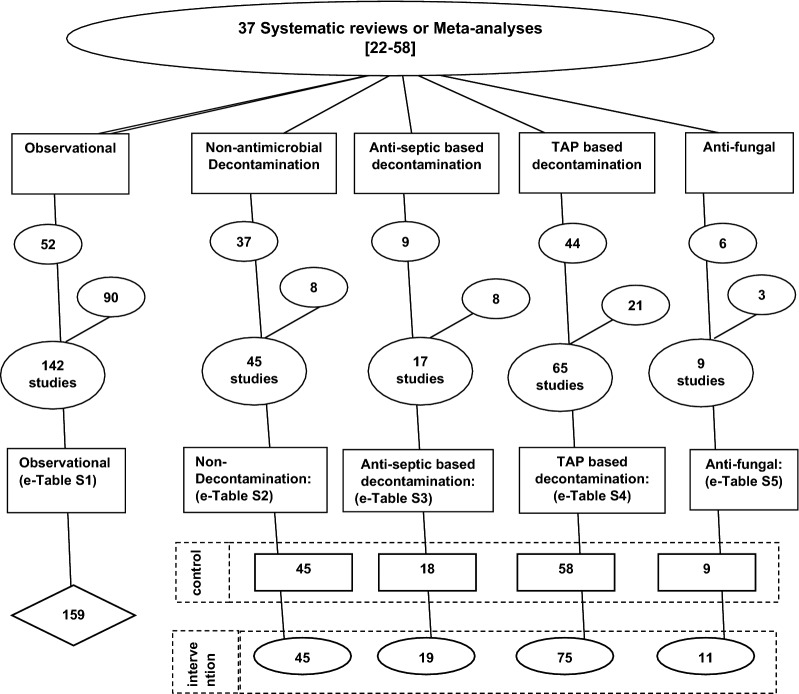
Search method, screening criteria and resulting classification of eligible studies and subsequent decant of component groups occured in the following steps; (1) An electronic search for systematic reviews in the Cochrane database using search terms; “ventilator associated pneumonia”, “mechanical ventilation”, “intensive care unit”, up to December 2021; (2) The systematic reviews were then searched for studies of patient populations requiring prolonged (> 24 h) ICU admission (3) The studies were triaged from the systematic reviews into one of five categories; studies in which there was no intervention (observational studies), studies of various non-decontamination methods or various methods of decontamination using either anti-septic, antibiotic (TAP) or antifungal prophylaxis. (4) Studies identified outside of these systematic reviews were included by a ‘snowball’ search for potentially eligible studies using the ‘related studies’ function in Google scholar. (5) the studies were reviewed for potentially eligible studies and screened against inclusion and exclusion criteria. Any duplicate or ineligible studies were removed and (6) The component groups were decanted from each study being control (rectangles), intervention (ovals) and observation (diamond) groups. Note; the total numbers do not tally as some systematic reviews provided studies in more than one category and some studies provided groups in more than one category and some studies have unequal numbers of control and interventions groups. *TAP*  topical antibiotic prophylaxis

Most of the studies had been cited within either one of the systematic reviews of The Cochrane review database or within additional systematic reviews found by snowball sampling using the ‘Related articles’ function within Google Scholar [37–59]. The snowball sampling also identified additional eligible studies. The study decant used here is as described previously [21] and is detailed in Fig. 2.

### Structural equation modelling

In these GSEM models, the *Candida* and Gram-positive bacterial infection data serve as the measurement components, the group level exposure parameters serve as the structural components and colonization with *Candida*, and individual Gram-positive bacteria*,* each represented as latent variables, link the structural and measurement components.

### Measurement components

The incidences of VAP with *Staphylococcus aureus* as well as the incidences of bacteremia with each of *Staphylococcus aureus,* CNS and *Enterococci* were extracted. As *Candida* is generally not counted as a cause of VAP, the count of *Candida* as a respiratory tract (RT *Candida*) isolate among patients with suspected VAP was recorded along with candidemia counts. Likewise, Enterococci and CNS are rarely recorded among VAP isolates. The use of Center for Disease control (CDC) criteria, being the requirement for at least two positive cultures for diagnosis of CNS bacteremia, was recorded. Counts for all subspecies of *Candida*, CNS and *Enterococci* were included. These were each expressed as a proportion using the number of patients with prolonged (> 24 h) ICU stay as the denominator. Note that colonization with *Candida*, *Staphylococcus aureus,* CNS and *Enterococcal* colonization are each derived within the models as latent variables and any colonisation data within any study was not used.

### Structural components

The following data were used to form the structural components of the models; year of study publication, origin from trauma ICU’s, whether more than 90% of patients of the group received more than 24 h of MV, and the mean (or median) length of ICU stay (LOS) for the group. In the extraction of MV percentages, if this was not stated for any group, the percentage receiving MV was assumed to be less than 90%. In the extraction of LOS data from the studies, surrogate measures including mean (or median) length of mechanical ventilation were taken if the length of LOS was not available.

Also, the presence of any of the following group wide risk factors for candidemia and invasive *Candida* infection were noted; liver transplantation or liver failure, use of parenteral nutrition, surgery for intestinal perforation, pancreatitis and being colonized with *Candida*, however that was defined. An anti-septic exposure included use of agents such as chlorhexidine, povidone-iodine and iseganan. All anti-septic exposures were included regardless of whether the application was to the oropharynx, by tooth-brushing or by body-wash.

Topical antibiotic prophylaxis (TAP) is defined here as the group wide application of topical antibiotic prophylaxis to the oropharynx or stomach without regard to the specific antibiotic constituents. The antibiotic-based regimens often use in addition protocolized parenteral antibiotic prophylaxis (PPAP), being the protocol driven group wide use of any parenteral antibiotic used on a prophylactic basis. Group wide exposure to anti-fungal prophylaxis was identified whether this was as a single agent (SAF) or used in combination with TAP within an SDD regimen, without regard to the specific anti-fungal agent.

#### Candidate SEM models

Four candidate GSEM models were developed in each of which colonization with *Candida* and the three individual Gram-positive bacteria constitute four latent variables. The models were constructed with and without the inclusion CRF as a predictor of bacterial colonisations, and with and without interaction terms between the colonization latent variables.

Because the observations are clustered by study, in each model a study identifier was used in order to generate a robust variance covariance matrix of the parameters of each coefficient estimate. The GSEM model with the lowest Akaike's information criterion (AIC) score was selected as having parsimony and optimal fit from among the candidate models using the ‘GSEM’ command in Stata (Stata 17, College Station Texas, USA) [60].

### Visual benchmarking

Scatter plots of the *Candida*, *Staphylococcus aureus,* CNS and *Enterococcal* infection incidence proportion data versus group mean LOS were generated to facilitate a visual survey of the entire data used in the analysis. To facilitate this visual survey, benchmarks, being the linear regression of logit transformed incidence proportion versus LOS derived for each isolate type was generated using the groups of the observational studies.

## Results

### Characteristics of the studies

Of the 283 studies identified by the search, 135 were sourced from 37 systematic reviews (Table 1) [22–58] with 148 found during previous searches or by snowball sampling (Fig. 2). Most studies were published between 1990 and 2010 and most had a group mean LOS exceeding ten days. A minority originated from either North American or trauma ICU’s. Twelve studies had more than one type of intervention groups and ten studies had no control group. The majority of groups from studies of infection prevention interventions had less than 150 patients per group versus more than 150 patients in the observational studies.

**Table 1:** Characteristics of studies

	Observational studies				
	(No intervention)	Non-econtamination	Anti-septic	Antibiotic	Anti-fungal
*Study characteristics*					
Sources^a^	Additional file 1: Table S1	Additional file 1: Table S2	Additional file 1: Table S3	Additional file 1: Table S4	Additional file 1: Table S5
Number of studies	146	46	18	66	9
Origin from systematic review^b^	52	37	9	44	6
Study publication year (range)	1987–2022	1987–2021	2000–2016	1984–2021	1994–2014
North American ICU’s^c^	29	9	8	6	2
Trauma ICUs^d^	25	9	3	13	0
*Group characteristics*					
Number of groups	167	92	39	137	20
Group mean LOS Mean (95% CI)	12.4 9.6–15.1	12.2 9.4–15.0	10.0 5.5–19.6	12.6 10.5–14.7	14.4 8.5–20.3
MV for > 48 h for < 90%^e^	39	0	17	35	12
PPAP use in control group^f^	0	0	0	9	0
CRF^g^	11	0	0	17	12
Use of CDC criteria^h^	26	0	5	19	0
Numbers of patients per control group; (median; IQR)^i^	290 123–660	75 61–143	132 31–347	55 38–84	47 23–51

Source data is presented in Additional file 1: Tables S1–S5. see Additional file 1 for additional tables, figures, and references

^a^Note, several studies had more than one control and or intervention group. Hence the number of groups does not equal the number of studies

^b^Studies that were sourced from 16 systematic reviews (references in Additional file 1)

^c^Study originating from an ICU in Canada  or the United States of America

^d^Trauma ICU arbitrarily defined as an ICU with more than 50% of admissions for trauma

^e^Groups for which less than 90% of patients were reported to receive > 48 h of MV

^f^Use of PPAP for control group patients. PPAP is protocolized parenteral antibiotic prophylaxis

^g^CRF is a term representing risk factors for either Candidemia or invasive *Candida* or patient groups selected on the basis of *Candida* colonization

^h^CDC is the Center for Disease control criteria for defining a CNS bacteremia as being at least two blood cultures positive for CNS

^i^Data is median and inter-quartile range (IQR)

Of the 460 groups from 283 studies, there were 25 groups from 13 studies with mean LOS less than 5 days including the largest of which (> 120,000 patients), being a study of targeted versus universal decontamination versus standard care [61].

There was a broad range of infection prevention exposures. The majority of data for anti-fungal exposures occurred in combination with TAP exposure within Antibiotic studies in the context of an SDD regimen for which the antifungal was topical amphotericin being used in 50 groups. Exposure to anti-fungal prophylaxis as a single agent occurred (within Anti-fungal studies) in only nine groups of studies of which five studies selected patients on the basis of risk factors for invasive candida infection. The TAP exposures included either topical polymyxin or a topical aminoglycoside or both in every case except four intervention groups. PPAP, most commonly a cephalosporin, was used within ten control groups and 48 intervention groups of TAP studies.

### Infection data

**Fig. 3:**
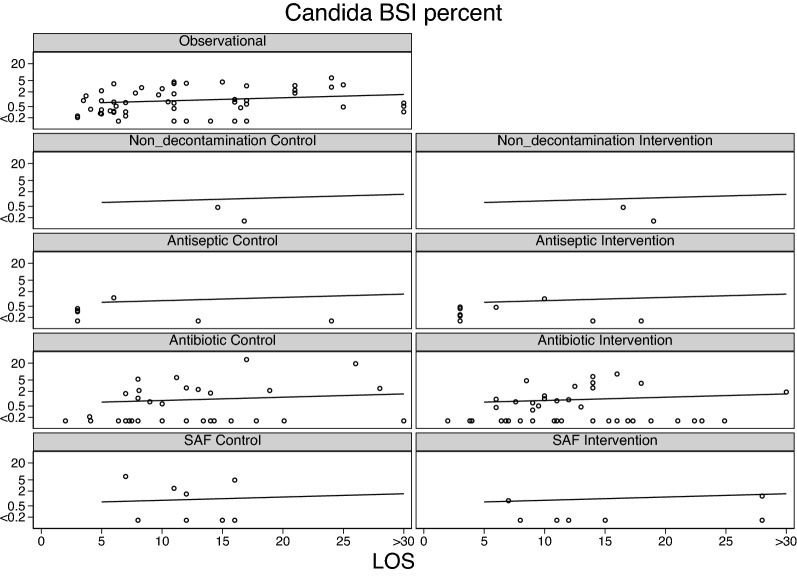
Candidemia (candida BSI) incidence proportion (logit scale) versus group mean length of stay (LOS; days) among categories of groups receiving either infection prevention interventions or control exposures. In each panel, the linear regression line derived from the observation studies is shown as a benchmark. Note that the candidemia incidence among the antifungal study intervention groups are asymmetrically distributed below the benchmark. The panels for RT candida incidence proportion versus group mean length of stay (LOS; days) are shown as Additional file 1

**Fig. 4:**
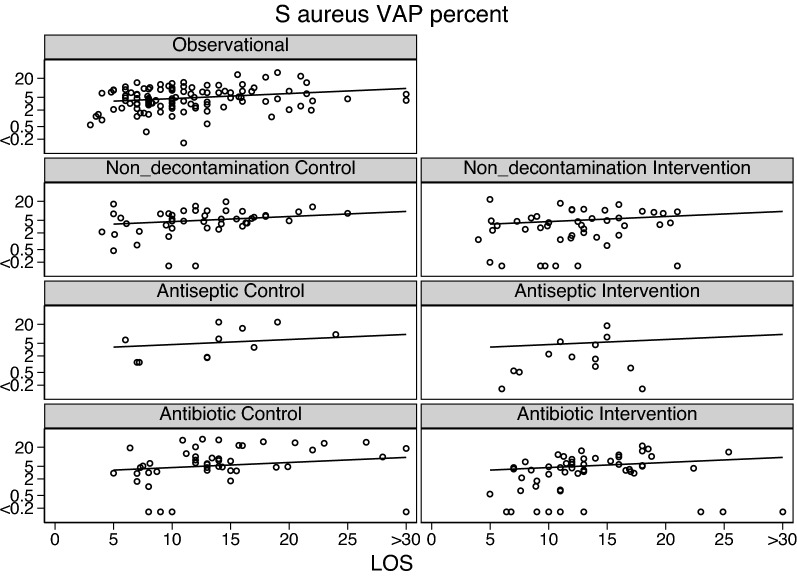
*S. aureus* VAP incidence proportion (logit scale) versus group mean length of stay (LOS; days) among categories of groups receiving either infection prevention interventions or control exposures. In each panel, the linear regression line derived from the observation studies is shown as a benchmark. Note that the *S. aureus* VAP incidences among the Antibiotic study control groups are asymmetrically distributed above the benchmark. The panels for the SAF studies are not shown as there were no observations in each

**Fig. 5:**
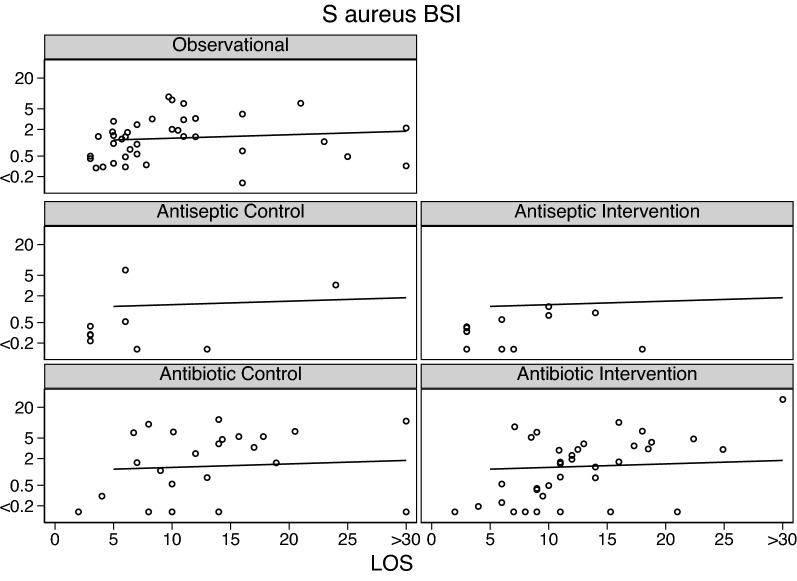
*S. aureus* BSI (bacteremia) incidence proportion (logit scale) versus group mean length of stay (LOS; days) among categories of groups receiving either infection prevention interventions or control exposures. In each panel, the linear regression line derived from the observation studies is shown as a benchmark. Note that the *S. aureus* BSI incidences among the antiseptic intervention groups are asymmetrically distributed below the benchmark. The panels for the SAF and anti-septic studies are not shown as there were fewer than three observations in each

**Fig. 6:**
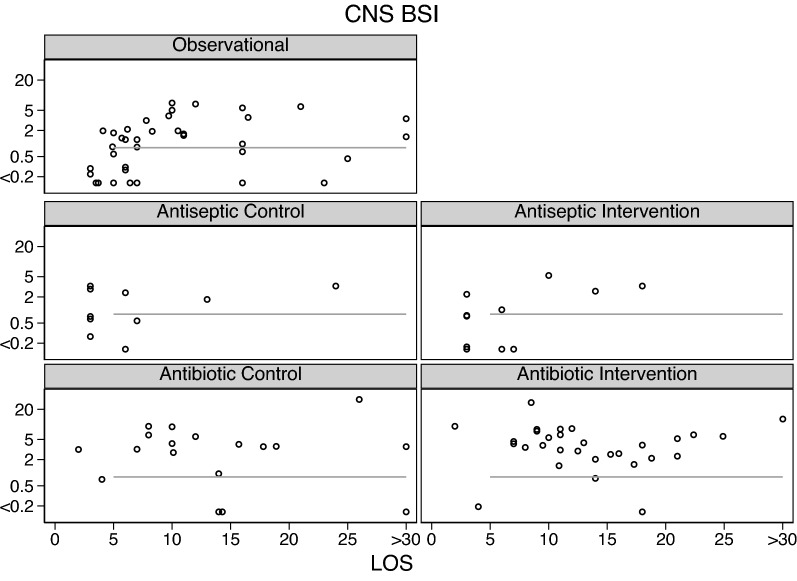
CNS BSI (bacteremia) incidence proportion (logit scale) versus group mean length of stay (LOS; days) among categories of groups receiving either infection prevention interventions or control exposures. In each panel, the linear regression line derived from the observation studies is shown as a benchmark. Note that the CNS BSI incidences among the control and intervention groups of the Antibiotic studies are asymmetrically distributed above the benchmark. The panels for the SAF and anti-septic studies are not shown as there were fewer than three observations in each

**Fig. 7:**
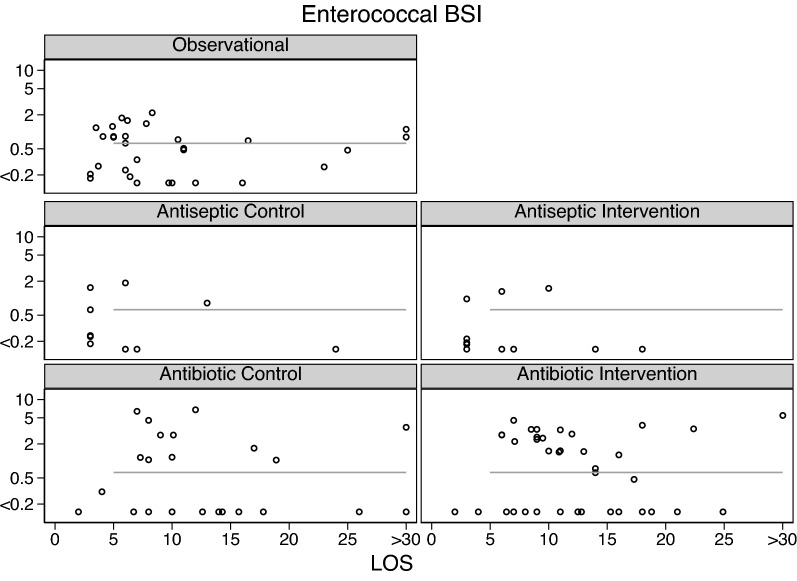
Enterococcal BSI (bacteremia) incidence proportion (logit scale) versus group mean length of stay (LOS; days) among categories of groups receiving either infection prevention interventions or control exposures. In each panel, the linear regression line derived from the observation studies is shown as a benchmark. Note that the Enterococcal BSI incidences among the control and intervention groups of the Antibiotic studies are asymmetrically distributed above the benchmark. The panels for the SAF and anti-septic studies are not shown as there were fewer than three observations in each

Across all intervention categories, the incidences for Candidemia (Fig. 3) and RT *Candida* (Additional file 1: Fig. S1), *S aureus* VAP (Fig. 4) and bacteremia (Fig. 5) and CNS (Fig. 6) and *Enterococcal* bacteremia (Fig. 7) varied in each case by > 100 fold and ranging approximately tenfold above and below the respective benchmarks. In each case, the incidence proportions mostly straddle the respective benchmarks with exceptions noted in the figure legends.

The mean control and intervention group incidences of VAP and bacteremia for each of *S aureus* (Fig. 2), CNS (Fig. 3) and *Enterococci* (Fig. 4) were generally similar to the benchmark derived from observational groups with the exception that *S aureus* VAP incidences among the control groups of Antibiotic studies were generally approximately five percentage points above the respective benchmark and the mean incidences of infection for each of CNS bacteremia (Fig. 3) and *Enterococcal* bacteremia (Fig. 4) among the control and intervention groups of Antibiotic studies were generally approximately two percentage points above the respective benchmarks.

### GSEM modelling

**Fig. 8:**
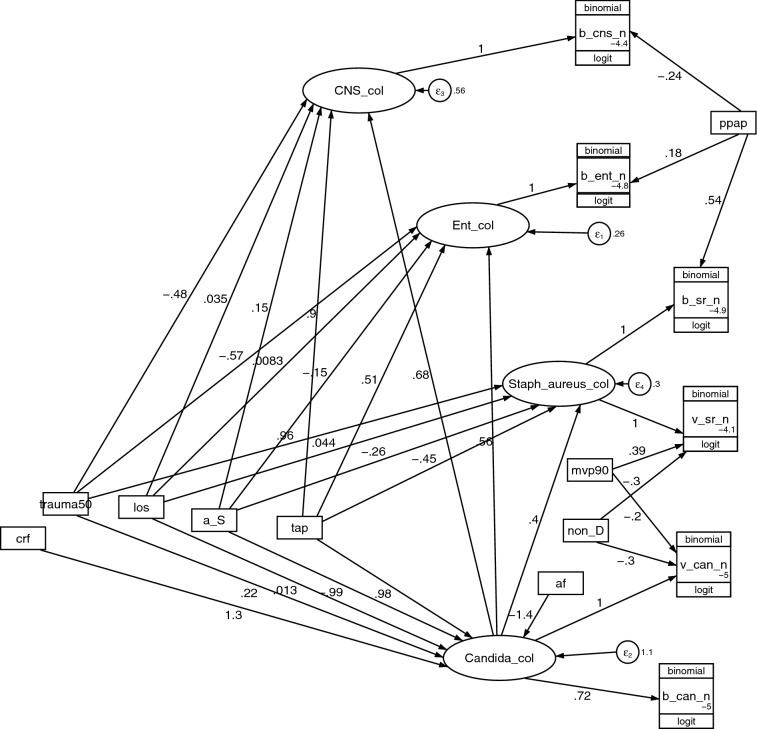
The optimal GSEM (model 4) representing the interaction term between *Candida* colonization and colonization with each of three Gram-positive bacteria. *Candida*_col, *S. aureus*_col, CNS_col and Ent_col (ovals) are latent variables representing *Candida*, *S. aureus*, CNS and *Enterococcal* colonization, respectively. The variables in rectangles are either continuous variables (los) representing ﻿mean or median length of ICU stay or binary predictor variables representing the group level exposure to the following; a trauma ICU setting (trauma50), topical anti-septic based prevention method (a_S), antibiotic based prevention method (tap), antifungal prophlyaxis (af), a non-decontamination based prevention method (non-D), use of mechanical ventialtion more for than 90% of the group (mvp90), or exposure to PPAP (ppap). The circles contain error terms (ɛ). The three-part boxes represent the count data for *Candida*, and *S aureus,* CNS and *Enterococci* as VAP (v_can_n, v_S aureus_n) and bacteremia (b_can_n, b_S aureus_n, b_cns_n, b_Ent_n) isolates. These counts are logit transformed with the total number of patients in each group as the denominator using the logit link function in the generalized model of the GSEM. The precursor models (Models 1–3) generated in the development of the optimal model are shown in Additional file 1: (Figs. S2–S4)

Four candidate GSEM models were evaluated for fit and parsimony (see Table 2; Fig. 8; Additional file 1: Figs. S2–S4). The optimal model, judged by AIC criteria, included an interaction term between the latent terms representing *Candida* colonization with each of the three Gram-positive bacteria colonization latent variables (Fig. 8). The size and statistical significance of this interaction term was similar in magnitude in each case. CRF predicted Candida colonization, as expected, but not bacterial colonization (Models 1–3; Additional file 1: Figs. S2–S4).

**Table 2:** Development of GSEM model

	Model 1	Model 2	Model 3	Model 4	
	Additional file 1: Fig. S2	Additional file 1: Fig. S3	Additional file 1: Fig. S4		Fig. 8
Factor^a–b^					95%CI
*Enterococcal colonization*^c^					
tap	0.62*	0.62*	0.62*	0.51**	0.12 to 0.89
a_S	− 0.46	− 0.45	− 0.46	− 0.15	− 0.57 to + 0.27
trauma50	− 0.31	− 0.31	− 0.31	− 0.57	− 1.2 to + 0.07
los	0.02	0.02	0.02	0.01	− 0.016 to + 0.03
crf	–	0.05	–	–	–
*Candida* col	–	–	–	0.56***	0.33 to 0.79
*CNS colonization*^d^					
tap	0.92**	0.93**	0.92**	0.90***	0.46 to 1.33
a_S	− 0.28	− 0.26	− 0.28	0.15	− 0.52 to 0.82
trauma50	− 0.22	− 0.18	− 0.22	− 0.48	− 0.99 to + 0.03
los	0.05*	0.05*	0.05*	0.03*	0.005 to 0.06
crf	–	0.26	–	–	–
*Candida* col	–	–	–	0.68***	0.34 to 1.0
*S aureus colonization*^e^					
tap	− 0.48***	− 0.49***	− 0.48***	− 0.45***	− 0.7 to − 0.2
a_S	− 0.65**	− 0.63**	− 0.65**	− 0.26	− 0.60 to 0.09
trauma50	1.01***	1.03***	1.01***	0.96***	0.67 to 1.23
los	0.05***	0.05***	0.05***	0.04***	0.02 to 0.06
crf	–	0.49	–	–	–
*Candida* col	–	–	–	0.40***	0.24 to 0.55
*Candida colonization*^gf^					
tap	0.80*	1.05**	1.05**	0.98**	0.35 to 1.61
a_S	− 1.16**	− 1.01*	− 1.01*	− 0.99**	− 1.66 to − 0.32
AF	− 1.17**	− 1.49***	− 1.49***	− 1.41***	− 2.1 to − 0.72
trauma50	− 0.02	0.19	0.19	0.22	− 0.48 to + 0.92
los	0.02	0.01	0.01	0.012	− 0.01 to + 0.04
crf	–	1.47**	1.47**	1.29**	0.43 to 2.15
*b_Ent_n*					
Enterococcal col	1	1	1	1	(constrained)
ppap	0.02	0.00	0.02	0.18	− 0.35 to 0.71
_cons	− 5.06***	− 5.06***	− 5.06***	− 4.82***	− 5.3 to − 4.3
*b_CNS_n*					
CNS col	1	1	1	1	(constrained)
cdc	− 0.14	− 0.12	− 0.07	− 0.11	− 0.71 to 0.61
ppap	− 0.07	− 0.17	− 0.07	− 0.24	− 0.84 to + 0.35
_cons	− 4.64***	− 4.66***	− 4.64***	− 4.40***	− 5 to − 3.9
*b_Sr_n*					
*S aureus* col	1.05***	1.04***	1.05***	1.03***	0.82 to 1.23
ppap	0.63*	0.57	0.63*	0.54	− 0.09 to + 1.17
_cons	− 5.00***	− 5.01***	− 5.00***	− 4.95***	− 5 to − 3.2
*b_ Candida _n*					
*Candida* col	0.81**	0.76**	0.76**	0.72***	0.3 to 1.14
_cons	− 5.00***	− 5.08***	− 5.08***	− 5.01***	− 5.3 to − 4.7
*v_ Sr _n*					
*S aureus* col	1	1	1	1	(constrained)
mvp90	0.45	0.48*	0.45	0.39	− 0.08 to + 0.86
non_D	− 0.29*	− 0.28*	− 0.29*	− 0.30*	− 0.58 to − 0.03
_cons	− 4.14***	− 4.19***	− 4.14***	− 4.09***	− 4.6 to − 3.6
*v_ Candida _n*					
*Candida* col	1	1	1	1	(constrained)
mvp90	− 0.45	− 0.12	− 0.12	− 0.20	− 1.01 to + 0.61
non_D	− 0.29	− 0.19	− 0.19	− 0.30	− 0.84 to + 0.24
_cons	− 4.73***	− 5.08***	− 5.08***	− 4.98***	− 5.8 to − 4.1
*Error terms*					
var (e. Ent col)	0.46*	0.13	0.58	0.21*	0.04 to 0.86
var (e. CNS col)	0.79***	0.43**	0.75*	0.29*	0.12 to 0.75
var (e. S aureus col)	0.45***	0.30***	0.45***	0.30***	0.21 to 0.43
var (e. *Candida* col)	1.48***	1.32***	1.49***	1.32***	0.87 to 1.9
*Model fit*^*g*^					
AIC	5747.13	5726.96	5724.73	5616.70	–
Groups(n)	450	450	450	450	–
Clusters (n)	274	274	274	274	–
Factors (n)	40	44	41	44	–

Shown in this table are all models toward developing the optimal model (model 4)

v _sr_n is the count of *Staphylococcus aureus* VAP; and v_can_n is the count of *Candida* isolates from patients with VAP; b_sr_n is the count of *Staphylococcus aureus* bacteremia; and b_can_n is the count of Candidemia; b_cns_n is the count of coagulative negative *Staphylococcus* bacteremia and b_ent_n is the count of *Enterococcal* bacteremia

PPAP is the group wide use of protocolized parenteral antibiotic prophylaxis; TAP is topical antibiotic prophylaxis; non-D is a non-decontamination intervention; year = year of study publication in units of ten (decade); Crf = Candidemia risk factor; Trauma50 are ICU's for which > 50% of admissions were for trauma; cdc is the use of CDC criteria for CNS bacteremia counts

^*^p < 0.05; **p < 0.01; ***p < 0.001

^a^MVP90 is use of mechanical ventilation by more than 90% of the group

^b^LOS is length of ICU stay

^c^Enterococcal colonization (Enterococcal col) is a latent variable

^d^CNS colonization (CNS col) is a latent variable

^e^S aureus colonization (S aureus col) is a latent variable

^f^*Candida* colonization (*Candida* col) is a latent variable

^g^Model fit; AIC is Akaike’s information criteria. This indicates model fit taking into account the statistical goodness of fit and the number of parameters in the model. Lower values of AIC indicate a better model fit. Groups is the number of patient groups; clusters is the number of studies; N is the number of parameters in the model

In the optimal model (model 4; Table 2; Fig. 8), the following exposures; TAP, trauma ICU admission, LOS and the interaction term with *Candida* colonization, displayed the strong associations with *S aureus* colonization. Exposure to TAP displayed positive associations with CNS colonization and Enterococcal colonization but a negative association with *S aureus* colonization (Table 2). The magnitude of these associations was similar in each case to that with the *Candida* colonization interaction term.

In all models, exposure to candidemia risk factors and TAP interventions displayed strong and consistent positive associations with *Candida* colonization and exposure to anti-septic, and antifungal interventions displayed strong and consistent negative associations with Candida colonization (Table 2).

## Discussion

There is a range of preclinical study evidence that suggests that interactions between *Candida* with other bacteria in the patient microbiome has the potential to promote invasive bacterial infections. The basis for the interaction may be molecular [7] or mechanical [8].

However, there are multiple obstacles to defining the clinical relevance of any interactions between *Candida* and bacteria in the patient microbiome. *Candida* colonization has several risk factors [1, 2] some of which, such as prolonged antibiotic exposure, are due to broad microbiome changes. *Candida* and bacterial colonization are problematic to quantify in relation to relevant body site, timing in relation to ICU stay, and whether as defined by viable counts versus biological activity. Various BSI, whether with Gram-positive bacteremias or candidemia are uncommon. Data arising from single center clinical studies, and especially so infection data, will exhibit dependency and also, will unlikely be generalizable. Finally, for *S aureus* bacteremia, being a relatively rare end point with a benchmark incidence of approximately 1.8%, even large multi-center studies may be underpowered to show any interaction with *Candida* colonization [62]. Moreover, other Gram-positive bacteria such as Enterococci are even less common than *S aureus* bacteremia. Finally, CNS bacteremia is variably defined in the studies with or without using CDC defining criteria.

Presumably as a result of these obstacles, attempts to define the clinical relevance of any interaction between *Candida* with other bacteria are scant and relate mostly to interactions with Gram negative bacteria. Conflicting results emerged mostly from single center studies which generally have fewer than 400 patients under study [63–69].

There is some evidence that the risk of VAP in association with *Pseudomonas aeruginosa* is more common in patients with *Candida* colonization [63] and that antifungal treatments can reduce this likelihood [64]. One study found that *Candida* colonization of the respiratory tract is associated with *Acinetobacter* VAP but not *Pseudomonas* VAP [65].

In contrast, other attempts to define the clinical relevance of any interaction between *Candida* with other bacteria through either retrospective studies of the association with anti-fungal use or through studies of either pre-emptive or intensified prophylactic anti-fungal treatment [66–68] have failed to resolve the question. Several have questioned the specificity of the association and whether any association is simply a reflection of confounding by illness severity [1, 69].

The approach here circumvents these obstacles by using data from > 450 patient groups from > 250 studies of infection prevention interventions among ICU patients as comprising a natural experiment. The various groups of these studies have been exposed to infection prevention interventions which, in conjunction with other exposures, are known to, either specifically or non-specifically, modify the patient microbiome. Of note, any one group here could experience multiple concurrent exposures such as concomitant CRF, TAP, PPAP, anti-fungal and a prolonged ICU-LOS. This is reflected in the wide range in incidences of infections across the > 250 groups here.

For example, using an SEM approach, the interaction between Candida colonization emerges as a key driver of *Pseudomonas* invasive infections among 279 studies of infection prevention methods among ICU patients. Moreover, the relative impacts of specific anti-fungal agents on *Pseudomonas* bacteraemia can be estimated and compared [21].

SEM is an established modelling technique. It has emerging applications in epidemiology [70], ecology [71], and critical care [72, 73] research for modelling the relationships between multiple simultaneously observed variables and potential (latent) variables in order to provide quantitative tests of any theoretical model proposed within the literature. The validity and inferred relationship of conceptual variables that cannot be directly quantified are testable by using latent variables within the model. GSEM allows generalized linear response functions in addition to the linear response functions allowed by SEM.

### Limitations

There are six key limitations to this analysis. Firstly, this analysis is a group level modelling of four latent variables being colonization with each of *Candida*, *Staphylococcus aureus,* CNS and *Enterococci*. These latent variables and the coefficients derived in the GSEM models are indicative and intended for internal reference only. They have no counterpart at the level of any one patient or study and cannot be directly measured. Specifically, colonization data within the studies was ignored in this analysis as the mere presence of colonizing Candida may not reflect the potential biological activity towards interactions with colonizing bacteria.

The GSEM analysis takes a structural rather than statistical approach to the question of any interactions between Candida and Gram-positive bacteria. The structural approach means that a limited number of conceptually key group level factors were entered mostly as simple binary variables into intentionally simplistic GSEM models. There was no ability nor purpose to adjust for the underlying patient level risk. The true relationships between exposures and outcomes will likely be center specific, complex, graded and with multiple expoure interactions. A conventional approach to estimating exposure effects requires analytic methods such as meta-analysis which, being based on an assumption of exchangability between control and intervention groups that randomized assignement of exposures provides, allows more precise effect size estimates for specific individual interventions under study. However, assumptions that outcomes in the control groups are not influenced by infection prevention interventions within an ICU setting are questionable [74]. Moreover, a randomized assignement of individual exposure to Candida colonization is neither an ethical nor a practical intervention outside of a natural experiement resulting from group level exposures to various ICU infection prevention interventions.

The second limitation is that there was considerable heterogeneity in the interventions, populations, and study designs among the studies here as the inclusion criteria for the various studies have been intentionally broadly specified. This breadth is both a strength, in that the breadth of the group exposures is the basis for the natural experiment here which serves as the basis for the research question central within the model. It is also a limitation, in that the associations for a group wide exposure may not equate to associations at the level of an individual patient exposure.

Thirdly, several assumptions have been made for studies that failed to report key exposure and outcome variables in the analysis. Missing LOS data and percent receiving MV have been imputed and there is missing infection count data which has not been imputed. The data is provided in sufficient detail in Additional file 1 to enable replication of the analysis.

Fourth, there are a large number of studies not included here because the required infection count data was not reported. However, the differences between control and intervention group mean infection incidences noted here in the scatter plots (Figs. 3, 4, 5, 6, 7, Additional file 1: Fig. S1) are similar in magnitude and direction to the summary effect sizes for each of the three broad categories of TAP, anti-septic and non-decontamination methods, against both overall VAP and against overall bacteremia which in turn are similar to prior published effect estimates sizes seen in systematic reviews of these interventions from which most of the studies examined here were derived [19–41].

Fifth, the various regimens of TAP, anti-septic and anti-fungal intervention that have been used within the various studies have been considered as similar within each category. This is a deliberate simplification as some, for example the anti-fungal regimens, target different body sites. Also, the duration of application of each regimen varied among the studies. On the other hand, a strength of this analysis is that it attempts to unpack the separate associations between the infection incidences and exposure to the various SDD components (TAP, PPAP, anti-fungal).

Finally, the literature search has been opportunistic rather than systematic. By using existing systematic reviews as a starting point, the key interventions can be readily identified and classified. As a consequence, the included studies have been predominately undertaken within first world country ICU’s. It is uncertain how representative this is of the microbiome for elsewhere in the world. There is some evidence that the bacteria that cause VAP vary in different parts of the world [75–77].

## Conclusion

GSEM modelling of interactions between colonization with *Candida* and three Gram-positive bacteria, each as latent variables provide support to the postulate that these interactions within the patient microbiome enhance the potential for invasive infections arising from colonizing bacteria. The magnitude of these interactions towards cause invasive infections may be similar in magnitude but contrary to that achieved with the infection prevention interventions. An interaction leading to enhanced invasive potential of Gram-positive bacteria might also account for the paradoxically high incidences among the groups of TAP studies.

## Supplementary Information

**Additional file 1: Table S1.** Observational studies (Benchmark groups). **Table S2.** Groups of non-decontamination studies. **Table S3.** Groups of Anti-septic studies. **Table S4.** Groups of Antibiotic (= TAP ± PPAP) studies. **Table S5.** Groups from single anti-fungal (SAF) studies. Reference list for included studies [Ref S1 - S283]. **Fig S1.** Candida RT count data. **Fig S2.** GSEM model. **Fig S3.** GSEM model. **Fig S4.** GSEM model.

## Data Availability

The datasets analysed during the current study are provided in Additional file 1.
