# Supplementary material for: Candida and the Gram-positive trio: testing the vibe in the ICU patient microbiome using structural equation modelling of literature derived data
Source: Emerg Themes Epidemiol. 2022 Aug 18;19:7. doi: 10.1186/s12982-022-00116-9 (PMC9387012; doi:10.1186/s12982-022-00116-9)

Candida and The Gram-positive trio: testing the vibe in the ICU patient microbiome using structural equation modelling of literature derived data.

James C Hurley, MB BS, D Med Sci, M Epi, PhD, FRACP

Supplemental file contents:

Table S1: Observational studies (Benchmark groups)	2 - 6
Table S2: Groups of non-decontamination studies	7- 10
Table S3: Groups of Anti-septic studies	11 – 12
Table S4: Groups of Antibiotic (= TAP ± PPAP) studies	13 – 18
Table S5: Groups from single anti-fungal (SAF) studies	19
References	20 – 36
Fig S1 Candida RT count data	37
Fig S2 GSEM model 1	38
Fig S3 GSEM model 2	39
Fig S4 GSEM model 3	40

Supplemental file abbreviations:

TAP = Topical antibiotic prophylaxis;

PPAP = Protocolized parenteral antibiotic prophylaxis;

SAF = single anti-fungal

Table S1: Observational studies (Benchmark groups) [a]

Author	Year	Ref	Notes	MV	LOS	Patients	VAP		Bacteremia			
				%	d	n	v_sr_n	v_can_n	b_sr_n	b_can_n	b_cns_n	b_ent_n
A'court	1993	1	T	100	12	150	4		5			
Alvarez-Lerma	1996	2		93	7	6494	102					
Antonelli	1994	3		70	17	124	10	1				
Apostolopoulou	2003	4		100	16	175	9					
Arumugam	2018	5	T	100	7	332	16					
Azoulay	2006	6		100	17	589				4		
Azoulay	2006	6	crf	100	17	214				2		
Bailly	2015	7		100	30	1491				11		
Badlesi	2020	8		64	7	246459			1490	851	2163	89
Bekaert	2011	9		100	8	4479	133					
Bercault	2001	10		100	26	1144		2				
Bercault_IHT	2005	11		100	9	118	2					
Bercault_noINT	2005	11	I	100	11	118	2					
Berrouane_all	1998	12		83	13	565	71					
Bloos	2022	13		82	11	169				7		
Bloos	2022	13		80	11	173				8		
Blot 45_64	2014	14		100	8	670	27					
Blot 65_74	2014	14		100	8	549	22					
Blot >74	2014	14		100	8	516	17					
Bochicchio	2004	15	T	100	13	678	50					
Bonten'94	1994	16		100	25	64	3					
Boots	2008	17		100	13	412	32	0				
Bornstain	2004	18		100	12	747	17					
Borzotta	1999	19		85	10	459				7		
Braun	1986	20		100	NS	66	6					
Bregeon	1997	21		100	NS	660	34	3				
Bronchard	2004	22	T	33	23	109	26					
Cade	1993	23		98	16	98	13	5	4	1	1†	0
Cavalcanti	2006	24		100	10	190	18	6				
Cenderero	1999	25		100	7	123	9	0				
Chaari	2015	26		100	8	175	6					
Charles	2005	27		75	14	36				0		
Charles	2005	27	crf	97	21	56				1		
Chastre	1998	28		100	14	243	18	7				
Chevret	1993	29		100	5	255	23	4				
Combes	2000	30	T	100	18	104	4					

Table S1 (continued): Observational studies (Benchmark groups)

Author	Year	Ref	Notes	MV	LOS	Patients	VAP		Bacteremia			
				%	d	n	v_sr_n	v_can_n	b_sr_n	b_can_n	b_cns_n	b_ent_n
Cook non-trauma	2010	31		100	8	2080	14	4				
Cook_trauma	2010	31	T	100	13	511	15	1				
Craven -medical	1988	32		100	6	277	9	1	4	0	0†	1
Craven -surgical	1988	32		100	6	521	12	5	10	3	11†	14
Daschner	1988	33		100	6	116	13	4				
De waele	2003	34	crf	79	16	28				0		
de_Latorre	1995	35		100	15	80	3	2				
de_Santis	2000	36		NS		713			13	2	17	9
de_Santis	2013	36		NS		1318			0	3	16	3
El-Masri	2004	37		NS	11	361			24	6	6†	2
Ensminger	2006	38	C	100	7	92	6					
Ertugrul	2006	39		100	10	100	12		9		4	0
Esteve	2007	40		80	17	404				0	23	0
Esteve	2007	40		78	16	395				2	15	3
Evans	2010	41		100	8	416	40					
Ewig	1999	42		100	10	48	5	1				
Fabian	1993	43	T	100	11	278	32					
Fagon	1989	44		100	13	567	17					
Ferreira	2015	45		94	5	2527						
Gacouin	2009	46		100	11	361	21					
Garcı´a -Garmendia	2001	47		46	5	2640			39	11	46	23
Garrouste -Orgeas	1997	48		100	11	86	13	1				
Garrouste -Orgeas	2006	49		75	11	3247			46	15	50†	17
George	1998	50		100	8	223	8	2				
Georges	2000	51		100	20	135	11					
Giamarellos-Bourboulis	2009	52	T	100	12	72		2	1	3	5†	0
Giard	2008	53		100	9	7236	193					
Gruson-95-96	2000	54		100		1004	67					
Gruson-97-98	2000	54	I	100		1029	54					
Gruson-99-01	2003	55		100		823	26					
Guérin	1997	56		100	19	260	3	0				
Gursel	2010	57		100	10	92	13	1				
Heyland	1999	58		100	7	1014	64	26		5		
Holzapfel_93	1993	59		100	10	300	8		6		22	6

Table S1 (continued): Observational studies (Benchmark groups)

Author	Year	Ref	Notes	MV	LOS	Patients	VAP		Bacteremia			
				%	d	n	v_sr_n	v_can_n	b_sr_n	b_can_n	b_cns_n	b_ent_n
Huang_1SC	2013	60		NS	3	15816			128	49	54†	42
Huang_1pre	2013	60		NS	3	23480			77	38	48†	33
Hugonnet	2007	61		100	6	936	55	40	40			
Hyllienmark	2007	62		100	4	221	2					
Hyllienmark	2013	63	T	42	3	135	11					
Ibáñez	2000	64		100	8	30	3					
Ibrahim'00	2000	65		100	5	1882	143	19				
Ibrahim'00	2000	66		69	11	4913			94	41	96†	38
Ibrahim'01	2001	67		56	9	880	36					
Jacobs	1990	68		100	15	24	2					
Jaillette	2011	69		100	15	439	22					
Jensen_HE	2015	70		66	5	604				24		
Jensen_SOC	2015	70		67	6	596				13		
Jimenez	1989	71		100	10	77	2					
Kautzky	2014	72		37	17	35		1		0		
Kautzky	2014	72	crf	57	24	30		1		2		
Ko	2013	73		100	23	1453			16		1	4
Kollef' 93	1993	74		100	7	277	9					
Kollef '95	1995	75		100	NS	314	17					
Kollef '95	1995	76		100	16	300	22					
Kollef '97	1997	77		100	8	521	25					
Kollef '97_post	1997	78	C I	90	4	327	4	0	1	3	0†	4
Kollef '97_pre	1997	78	C	90	4	353	5	1	5	5	0†	1
Koss– N	2001	79		100	11	87	3	10				
Koss– P	2001	79	I	100	11	66	4	3				
Kunac	2014	80		100	NS	716	62		6		0†	
Laggner	1989	81		100	11	32	0	0	1	0		
Lambert	2011	82		NS	5	119699			462			
Laupland	2002	83		NS	5	1017			18	3	9†	3
Laupland	2004	84		84	5	4473			45	19	28†	3
León	2006	85	crf	95	21	1699				58		
León	2009	86	crf	91	17	1107				37		
León	2016	87	crf	84	15	233				11		
Lepelletier	2010	88	T	100	18	161	34					
Li	2012	89		17	12	29		1		0		
Li	2012	89	crf	40	25	82		10		3		

Table S1 (continued): Observational studies (Benchmark groups)

Author	Year	Ref	Notes	MV	LOS	Patients	VAP		Bacteremia			
				%	d	n	v_sr_n	v_can_n	b_sr_n	b_can_n	b_cns_n	b_ent_n
Luna	2003	90		100	8	427	19	2				
Luyt	2005	91		100	NS	290	12					
Magnason	2008	92		100	8	280	1	0	1	5	9†	4
Mahul	1992	93		100	22	145	10	3				
Makris	2011	94	I	100	22	152	3					
Markowicz	2000	95		100	10	744	74					
Massart	2021	96	T	70	6	2464			13	8	7	6
Memish	2000	97		100	11	202	16	4				
Michel	2005	98		100	11	299	12					
Mitsogianni	2011	99		NS	16	124			0	1	0†	
Mitsogianni	2010	99		NS	16	143			1	0	1†	
Moine	2002	100		80	14	764	19	2				
Montecalvo	1992	101		100	10	38			3	1	2†	0
Myny	2005	102		100	4	385	27					
Nguile -Makao	2010	103		100	7	2873	89					
Nielsen	1992	104		100	5	242	5					
Nseir	2005	105		100	10	1241	15					
Nseir	2007	106	crf	100	24	102				3		
Orsi	2007	107		98	36	1741			37	10	61†	20
Orsi	2012	107		100	34	1165			4	4	17†	10
Osmon	2003	108		72	8	893			29	26	17†	19
Outcomerea	2019	109		100	8	7735	258					
Papazian	1996	110		100	10	586	20					
Petri	1997	111	crf	95	11	409				3		
Potgieter	1987	112		78	9	250	23	5				
Prowle	2011	113		69	6	6339			88	51	80†	56
Ramirez	2016	114		100	13	440	8	0				
Rello'91	1991	115		100	8	264	15	1				
Rello'92	1992	116		80	8	208	22					
Rello'92	1992	117		67	9	161	21					
Rello'94	1994	118		72	NS	1650			16	4	21	6
Rello'02	2002	119		100	8	9080	143					
Rello'03	2003	120		100	20	99	2	0				
Resende	2013	121		100	22	126	5	0				
Reusser	1989	122		100	7	40	6		1		0	0
Rincón -Ferrari	2004	123		100	10	310	27					

Table S1 (continued): Observational studies (Benchmark groups)

Author	Year	Ref	Notes	MV	LOS	Patients	VAP		Bacteremia			
				%	d	n	v_sr_n	v_can_n	b_sr_n	b_can_n	b_cns_n	b_ent_n
Rodrigues	2009	124		100	10	133	11	2	2			
Rodriguez	1991	125	T	100	14	294	37					
Ruiz-Santana	1987	126		100	7	1005	12	1	1			
Salata	1987	127		100	11	51	2	1	1			
Shahin	2013	128		100	10	267	6	3				
Sofianou	2000	129		100	36	198	13					
Stéphan	2006	130	T	100	16	175	43					
Stolcin	2020	131		100	6	930	47	5	11	12	11†	23
Stolcin	2020	131		27	6	3388						
Tan	2016	132		100	13	618	23					
Tejada -Artigas	2001	133		100	12	103	11			0		
Thompson	2008	134		NS	6	4270			74	24	89	68
Timsit	1996	135		100	19	387	18					
Torres	1990	136		100	3	322	2					
Trouillet	1998	137		100	NS	498	52					
Urli	2002	138		95	21	178	40	1	12	4	11†	
Valles	2007	139		100	22	60	9					
Vanhems	2011	140		100	6	3387	137					
Verhamme	2007	141		84	8	4000	56	6				
Violan	1998	142		100	16	314	26					
Warren	2001	143		28	4	3163			3	4	18†	8
Woske	2001	144		100	19	103	29					
Xie	2011	145		100	25	4155	92	88				
Zahar	2009	146		100	10	1233	51					

Table S1 footNotes

Notes; T = Data originating from a study for which the majority of ICU admission were for trauma; C = cardio-thoracic ICU; I = Infection control intervention to entire ICU; crf = group wide candidemia risk factor

MV = percentage of group receiving mechanical ventilation; NS – Not stated; LOS is mean or median length of ICU stay; The ICU-LOS is the ICU length of stay. This is based on surrogate measures including mean (or median) length of MV were taken if the length of ICU LOS was not available in order to generate broad categories of ICU stay of <5 days, 5 to 10 days and >10 days

v _sr_n is the count of *Staphylococcus aureus* VAP; and v_can_n is the count of *Candida* isolates from patients with VAP; b_sr_n is the count of *Staphylococcus aureus* bacteremia; and b_can_n is the count of Candidemia; b_cns_n is the count of coagulative negative *Staphylococcus* bacteremia (those studies using CDC criteria are indicate by †) and b_ent_n is the count of *Enterococcal* bacteremia.

Table S2: Groups of non decontamination studies [a]

Author	Year	Ref	Notes	MV	LOS	Patients	VAP		Bacteremia			
				%	d	n	v_sr_n	v_can_n	b_sr_n	b_can_n	b_cns_n	b_ent_n
Acosta -escribano	2010	147	T	100	18	54	4	1				
Acosta -escribano	2010	147	T	100	16	50	8	0				
Bonten '95	1995	148		100	19	67	4			0		
Bonten '95	1995	148		100	17	74	7			0		
Cook	1998	149		100	13	604	36	11				
Cook	1998	149		100	14	596	44	19				
Daumal	1999	150		100	6	174	7	2				
Daumal	1999	150		100	7	187	9	6				
Djedaini	1995	151		100	10	68	2	1				
Djedaini	1995	151		100	9	61	0	0				
Drakulovic	1999	152		100	10	39	0	0				
Drakulovic	1999	152		100	9	47	4	0				
Dreyfuss	1991	153		100	10	28	1					
Dreyfuss	1991	153		100	13	35	2					
Dreyfuss	1995	154		100	10	70	2					
Dreyfuss	1995	154		100	13	61	0					
Driks	1987	155		100	14	61	0	0				
Driks	1987	155		100	11	69	4	0				
Forestier	2008	156		100	13	106	11					
Forestier	2008	156		100	13	102	12					
Francois	2021	157		100	21	100	26		7			
Francois	2021	157		100	19	96	17		3			
Heyland	1999	158		100	12	49	1	0				
Heyland	1999	158		100	13	46	0	0				
Holzapfel_C	1999	159		100	15	200	21	3	2	1	29	9
Holzapfel_I	1999	159		100	17	199	7	1	2	1	22	5
Kappstein	1991	160		100	5	49	11	3				
Kappstein	1991	160		100	5	55	9	7				
Kirschenbaum	2002	161		100	21	20	1					
Kirschenbaum	2002	161		100	20	17	0					
Kirton	1997	162		100	NS	140	6					
Kirton	1997	162		100	NS	140	6					
Knight	2009	163		100	7	130	0	0				
Knight	2009	163		100	6	129	1	0				

Table S2 (continued): Groups of non decontamination studies [a]

Author	Year	Ref	Notes	MV	LOS	Patients	VAP		Bacteremia			
				%	d	n	v_sr_n	v_can_n	b_sr_n	b_can_n	b_cns_n	b_ent_n
Kollef	2008	164		100	4	743	16	7				
Kollef _silverETT	2008	164		100	4	766	9	5				
Lacherade	2005	165		100	25	185	18	0				
Lacherade	2005	165		100	21	184	16	2				
Lacherade	2010	166		100	11	164	8					
Lacherade	2010	166		100	11	169	2					
Laueny	2014	167		100	17	98	17	0				
Laueny	2014	167		100	11	91	6	0				
Lorente '03	2003	168		100	18	114	7	14				
Lorente '03	2003	168		100	16	116	8	10				
Lorente '04	2004	169		100	16	161	14	2				
Lorente '04	2004	169		100	20	143	6	3				
Lorente '05	2006	170		100	13	210	10	2				
Lorente '05	2006	170		100	13	233	11	1				
Lorente '06a	2005	171		100	10	236	8	1				
Lorente '06a	2005	171		100	10	221	8	1				
Lorente '06b	2006	172		100	NS	53	2	0				
Lorente '06b	2006	172		100	NS	51	5	0				
Lorente '07	2007	173		100	16	140	2	0				
Lorente '07	2007	173		100	14	140	8	0				
Lorente'14	2014	174		100	16	150	5					
Lorente'14	2014	174		100	15	134	1					
Manzano	2008	175		100	12	63	9					
Manzano	2008	175		100	9	64	4					
Martin	1993	176		100	10	65	0	1				
Martin	1993	176		100	10	66	1	0				
Morrow	2010	177		100	15	73	8	0				
Morrow	2010	177		100	15	73	14	1				
Nseir	2011	178		100	10	61	3					
Nseir	2011	178		100	10	61	1					
Pickworth	1993	179	T	100	3	44	1	0				
Pneumatikos	2006	180		100	16	39	2	0				
Pneumatikos	2006	180		100	15	40	4	0				
Prod'hom_A	1994	181		100	6	81	5					
Prod'hom_R	1994	181		100	5	80	4					
Prod'hom_S	1994	181		100	5	83	2					

Table S2 (continued): Groups of non decontamination studies [a]

Author	Year	Ref	Notes	MV	LOS	Patients	VAP		Bacteremia			
				%	d	n	v_sr_n	v_can_n	b_sr_n	b_can_n	b_cns_n	b_ent_n
Reigneir	2013	182		100	10	227	10					
Reigneir	2013	182		100	10	222	17					
Rumbak	2004	183		100	5	60	1					
Rumbak	2004	183		100	16	60	5					
Ryan_C	1993	184		100	5	56	1					
Ryan_S	1993	184		100	6	58	2					
Smulders	2002	185		100	14	75	3	1				
Smulders	2002	185		100	12	75	1	0				
Staudinger	2010	186		100	14	75	2	2				
Staudinger	2010	186		100	8	75	2	0				
Thomachot	1998	187		100	12	66	7	0				
Thomachot	1998	187		100	12	70	8	0				
Thomachot	1999	188		100	11	77	8	0				
Thomachot	1999	188		100	12	63	7	0				
Thomachot	2002	189		100	9	71	5					
Thomachot	2002	189		100	9	84	7					
Valencia	2007	190		100	13	69	2					
Valencia	2007	190		100	13	73	2					
Walaszek	2017	191		100	5	804	4	5				
Walaszek	2017	191		100	5	1003	2	1				
Zeng	2016	192		100	22	118	12	2				
Zeng	2016	192		100	18	117	16	4				

Table S2 footNotes

Notes; T = Data originating from a study for which the majority of ICU admission were for trauma; C = cardio-thoracic ICU; I = Infection control intervention to entire ICU; crf = group wide candidemia risk factor

MV = percentage of group receiving mechanical ventilation; NS – Not stated; LOS is mean or median length of ICU stay, The ICU-LOS is the ICU length of stay. This is based on surrogate measures including mean (or median) length of MV were taken if the length of ICU LOS was not available in order to generate broad categories of ICU stay of <5 days, 5 to 10 days and >10 days

v _sr_n is the count of *Staphylococcus aureus* VAP; and v_can_n is the count of *Candida* isolates from patients with VAP; b_sr_n is the count of *Staphylococcus aureus* bacteremia; and b_can_n is the count of Candidemia; b_cns_n is the count of coagulative negative *Staphylococcus* bacteremia (those studies using CDC criteria are indicate by †) and b_ent_n is the count of *Enterococcal* bacteremia.

Table S3: Groups of anti-septic studies [a]

Author & regimen	Year	Ref	Notes	MV	LOS	Patients	VAP		Bacteremia			
				%	d	n	v_sr_n	v_can_n	b_sr_n	b_can_n	b_cns_n	b_ent_n
Bellissimo-Rodrigues	2014	193		76	11	127		1				
Bellissimo-Rodrigues Chlx	2014	193		77	11	127		0				
Bleasdale	2007	194		35	3	445			1	1	15†	
Bleasdale Chlx	2007	194		36	3	391			0	0	3†	
Cabov	2010	195		57	6	30	2		2		0	0
Cabov	2010	195		77	6	30	0		0		0	0
Caruso	2009	196		100	17	132	5	3				
Caruso Sal	2009	196		100	17	130	1	0				
Climo	2013	197		NS	6	1398			42	16	34†	26
Climo Chlx(BW)	2013	197		NS	6	1410			24	7	15†	19
Fourrier	2000	198		100	24	30	3	1	1	0	1†	0
Fourrier Chlx	2000	198		100	18	30	0	0	0	0	1†	0
Fourrier	2005	199		100	13	114	2	0	0	0	2†	1
Fourrier Chlx	2005	199		100	14	114	1	0	1	0	3†	0
Huang_2pre	2013	60		NS	3	15218			70	56	43†	37
Huang_3pre	2013	60		NS	3	17356			80	59	116†	44
Huang_2TD	2013	60		NS	3	24752			106	63	42†	45
Huang_3UD	2013	60		NS	3	26024			92	62	36†	50
Koeman	2006	200		100	13	130	5	1				
Koeman -Chlx	2006	200		100	14	127	2	3				
Koeman ChlxC	2006	200		100	13	128	5	4				
Kollef	2006	201		100	13	347	25	6				
Kollef Iseganan	2006	201		100	14	362	17	0				
Lorente	2012	202		100	14	219	4	0				
Lorente Chlx	2012	202		100	13	217	4	0				
Milstone	2013	203		NS	3	1326			4	6	38	9
Milstone	2013	203		NS	3	667			3	3	15	1
Mori	2006	204		100	12	414	5	2				
Mori PVI	2006	204		100	7	1248	7	1				
Noto	2015	205		NS	8	4852			15	6	37†	9
Noto Chlx(BW)	2015	205		NS	3	4488			16	2	36†	10

Table S3 (continued): Groups of anti-septic studies [a]

Author & regimen	Year	Ref	Notes	MV	LOS	Patients	VAP		Bacteremia			
				%	d	n	v_sr_n	v_can_n	b_sr_n	b_can_n	b_cns_n	b_ent_n
Seguin	2006	206		100	19	31	7					
Seguin	2006	206		100	14	31	7					
Seguin-PVI	2006	206	T	100	15	36	3					
Seguin	2014	207		100	16	72	11					
Seguin-PVI	2014	207		100	15	78	14					
Swan	2016	208		57	7	164	2	1	0		1	0
Swan Chlx(BW)	2016	208		69	7	161	1	0	0		0†	0
Wittekamp Chlx	2018	209		100	10	2108			25	22	117	32

Table S3: Footnotes

Notes; T = Data originating from a study for which the majority of ICU admission were for trauma; C = cardio-thoracic ICU; I = Infection control intervention to entire ICU; crf = group wide candidemia risk factor

MV = percentage of group receiving mechanical ventilation; NS – Not stated; LOS is mean or median length of ICU stay; The ICU-LOS is the ICU length of stay. This is based on surrogate measures including mean (or median) length of MV were taken if the length of ICU LOS was not available in order to generate broad categories of ICU stay of <5 days, 5 to 10 days and >10 days

v _sr_n is the count of *Staphylococcus aureus* VAP; and v_can_n is the count of *Candida* isolates from patients with VAP; b_sr_n is the count of *Staphylococcus aureus* bacteremia; and b_can_n is the count of Candidemia; b_cns_n is the count of coagulative negative *Staphylococcus* bacteremia (those studies using CDC criteria are indicate by †) and b_ent_n is the count of *Enterococcal* bacteremia.

Intervention regimens abbreviations

Chlx = chlorhexidine; Chlx BW = chlorhexidine body wash; ChC = chlorhexidine and colisitn; TD = targetted decolonization; UD = universal decolonization; PVI = povidone iodine; CC = concurrent control; SC = saline control; iseganan, is a synthetic variant of a porcine protegrin, which is a natural antibiotic peptide released by neutrophils in response to invasion by microbes [Kollef 2006].

Table S4: Groups of studies of topical antibiotics [a]

Author & regimen	Year	Ref	Notes	MV	LOS	Patients	VAP		Bacteremia			
				%	d	n	v_sr_n	v_can_n	b_sr_n	b_can_n	b_cns_n	b_ent_n
Groups from NCC studies												
Bergmans	2001	210		100	12	61	5	1				
Bonten	1994	211		91	13	54	2					
Camus	2014	212		28	4	925			3	2	7†	3
Camus PTA	2014	212		28	4	1022			2	1	2†	1
De la Court	2021	213		NS	NS	1236			12	11	34	47
De la Court	2021	213		NS	NS	722			7	4	24	25
de Smet	2009	214		88	9	1990			22	16		55
de Smet PTA-Ctx	2009	214		93	9	2045			9	8		48
de Smet PTA	2009	214		94	9	1904			9	14		49
Garbino PNeV	2002	215		100	9	204	8	2	11	10	52†	7
Godard	1990	216		80	13	84	4			2		
Godard PT	1990	216		81	11	97	0			0		
Hartenauer Ctx	1991	217		100	14	101			3		1†	0
Hartenauer PTA-Ctx	1991	217		100	13	99			4		3†	0
Hjortrup CefTMyco	1997	218	crf	100	NS	150	14	11	2	4	3	1
Konrad	1989	219		100	NS	83	4					
Konrad PTA-Ctx	1989	219		100	NS	82	2					
Landelle	2018	220		100	9	291	10					
Landelle PTNy	2018	220		100	8	413	9					
Landelle PTNy	2018	220		100	9	356	4					
Ledingham	1988	221		60	5	161	5					
Ledingham PTA-Ctx	1988	221		60	5	163	1					
Leone PTA-Cef	2002	222		100	12	324	23					
Mathieu	2020	223	T	100	NS	199				0		
Mathieu	2020	223	T	100	NS	248				1		
Nardi	1990	224		100	13	50	3					
Nardi PTA	1990	224		100	12	47	3					
Nardi PTA	2001	225	T	100	12	104	9	1	2	1	9	3
Nardi PTAM	2001	225	T	100	11	119	1	0	2	0	10	4
Ong PTA-Ctx	2015	226		87	10	3080			10	17	122	76

Table S4 (continued): Groups of studies of topical antibiotics [a]

Author & regimen	Year	Ref	Notes	MV	LOS	Patients	VAP		Bacteremia			
				%	d	n	v_sr_n	v_can_n	b_sr_n	b_can_n	b_cns_n	b_ent_n
Oostdijk PTA-Ctx	2014	227		51	6	5483			13	26		151
Oostdijk PTA	2014	227		52	6	5508			32	55		154
Rouby E	1994	228		100	18	251	14					
Rouby P	1994	228		100	12	347	21					
Silvestri PTA-Ctx	1999	229	e	100	9	117	2	0	8	0	9†	4
Steffen PTNy-Ctx	1994	230	crf	100	14	127			1	0	1	1
Stoutenbeek	1984	231		100	14	59	18	0	0	1	0†	0
Stoutenbeek PTA-Ctx	1984	231		100	11	63	0	0	0	0	2†	0
Stoutenbeek	1987	232		100	14	59	3	0	7		0†	0
Stoutenbeek PTA	1987	232		100	18	42	9	0	3		0†	0
Stoutenbeek PTA-Ctx	1987	232		100	11	63	3	0	0	0	2†	0
Valles	2013	233		100	15	58	1					
Valles Ctx	2013	233		100	10	71	2					
Veelo PTA-Ctx	2008	234		100	17	231	8					
Winter	1992	235		92	7	84	4	0		0		1
Wittekamp	2018	209		100	10	2251			13	15	97	27
Wittekamp PTNy	2018	209		100	10	2224			12	23	127	34
Wittekamp PTNy	2018	209		100	11	2082			17	18	135	32
Groups from CC studies												
Abele-Horn	1997	236	T	100	22	30	5					
Abele-Horn PTA-Ctx	1997	236	T	100	18	58	9					
Acquarolo	2005	237		100	13	19	6	0				0
Acquarolo Ampsul	2005	237		100	13	19	3	0				0
Aerdts	1991	238		100	28	39	4	2		1		
Aerdts PNoA-Ctx	1991	238		100	23	17	0	0		0		
Bergmans	2001	210		100	13	78	6	3				
Bergmans PGV	2001	210		100	15	87	3	1				
Bion	1991	239	crf	50	2	31		2	0	0	1	0
Bion PTA-Ctx	1991	239	crf	50	2	21		3	0	0	2	0

Table S4 (continued): Groups of studies of topical antibiotics [a]

Author & regimen	Year	Ref	Notes	MV	LOS	Patients	VAP		Bacteremia			
				%	d	n	v_sr_n	v_can_n	b_sr_n	b_can_n	b_cns_n	b_ent_n
Blair	1991	240		93	8	130	7	1		0		
Blair PTA-Ctx	1991	240		93	8	126	1	0		1		
Blaise	1994	241	crf	33	7	45			3			0
Blaise Ofl	1994	241	crf	37	7	46			4			1
Bonten	1994	211		86	9	21	0					
Bonten PTA	1994	211		100	13	22	0					
Bouza	2013	242	C	100	12	38	4	0				
Bouza Lnz-Mrp	2013	242	C	100	10	40	2	0				
Camus	2005	243		100	13	126	8		1			
Camus MCh	2005	243		100	10	130	3		1			
Camus PT	2005	243		100	12	130	6		3			
Camus PT&MCh	2005	243		100	11	129	1		2			
Cerra	1992	244	e	26	26	21				4	6†	0
Cerra NoNy	1992	244	e	18	18	25				1	1†	1
Cockerill	1992	245		85	12	75				2		
Cockerill PGNy-Ctx	1992	245		85	10	75				1		
de la Cal	2005	246	T	80	34	54	10	0	6	0	2†	2
de la Cal PTA-Ctx	2005	246	T	74	31	53	0	2	14	1	7†	3
Ferrer Ctx	1994	247		100	14	41	2	2	2	0	0	0
Ferrer PTA-Ctx	1994	247		100	15	39	3	0	0	0	1	0
Flaherty	1990	248	C	40	4	56				0		
Flaherty PGA-Ctx	1990	248	C	40	4	51				0		
Garbino_ PNeV	2004	249		100	14	71		1		3		
Garbino_ PNeV	2004	249	crf	100	14	29		0		2		
Gaussorgues	1991	250		100	17	59		1		1		
Gaussorgues PGA	1991	250		100	16	59		0		0		
Georges	1994	251		100	7	33	1	0		0		
Georges PNeA	1994	251		100	7	31	0	0		0		

Table S4 (continued): Groups of studies of topical antibiotics [a]

Author & regimen	Year	Ref	Notes	MV	LOS	Patients	VAP		Bacteremia			
				%	d	n	v_sr_n	v_can_n	b_sr_n	b_can_n	b_cns_n	b_ent_n
Hammond Ctx	1994	252		100	14	33	3					
Hammond PTA-Ctx	1994	252		100	16	39	4					
Hellinger	2002	253	crf	NS	21	43			0	0	1†	0
Hellinger PGNy	2002	253	crf	NS	21	37			0	0	2†	0
Jacobs	1992	254		100	10	43	0	0	0	0	4	0
Jacobs PTA-Ctx	1992	254		100	9	36	0	0	0	0	3	0
Karvouniaris	2015	255		100	13	84	4					
Karvouniaris P	2015	255		100	16	84	5					
Kerver	1988	256		100	20	47		0		0		
Kerver PTA-Ctx	1988	256		100	17	49		0		0		
Korinek	1993	257		100	27	60	16	0				
Korinek PTA-V	1993	257		100	25	63	9	0				
Laggner	1994	258		100	32	34	0	0	0	0	0	0
Laggner GA	1994	258		100	25	33	0	0	1	0	2	0
Langlois -Karaga	1995	259		100	11	50	15					
Langlois -Karaga PGA	1995	259		100	11	47	5					
Palomar	1997	260		100	6	42	8	0		0		
Palomar PTA-Ctx	1997	260		100	8	41	5	0		0		
Palomar Ctx	1997	260		100	11	46	3			1		
Quinio	1995	261	T	100	16	72	16	0	4	0	3†	0
Quinio PGA	1995	261	T	100	16	76	9	0	8	0	2†	1
Rimola	1985	262	crf	NS	10	72			5		2	2
Rimola PGVNy	1985	262	crf	NS	11	68			2		1	1
Rocha	1992	263	T	100	18	54	15		3	0	2†	0
Rocha PTA-Ctx	1992	263	T	100	19	47	5		2	0	1†	0
Rodríguez -Roldán	1990	264		100	12	15	1					
Rodríguez -Roldán PTNeA	1990	264		100	10	14	0					

Table S4 (continued): Groups of studies of topical antibiotics [a]

Author & regimen	Year	Ref	Notes	MV	LOS	Patients	VAP		Bacteremia			
				%	d	n	v_sr_n	v_can_n	b_sr_n	b_can_n	b_cns_n	b_ent_n
Rolando	1996	265	crf	73	NS	61	1	1	1	1	2[†]	4
Rolando PTA	1996	265	crf	73	NS	47	2	1	0	0	2[†]	0
Rolando	1993	266	crf	75	8	31	1	3	3	0	2[†]	0
Rolando PTAM-Cfu	1993	266	crf	75	8	28	2	0	0	0	1[†]	0
Sanchez-Garcia	1998	267		100	20	140	7	0				
Sanchez-Garcia PTA-Ctx	1998	267		100	17	131	5	0				
Sirvent	1997	268		100	16	50	11	0				
Sirvent -Cef	1997	268		100	13	50	3	0				
Smith	1993	269	crf	100	8	18		1		1		
Smith PTA-Ctx	1993	269	crf	100	7	18		0		0		
Stoutenbeek	2007	270	T	100	12	200	40	21	5	0	12	14
Stoutenbeek PTA-Ctx	2007	270	T	100	13	201	18	6	8	1	9	3
Ulrich	1989	271		83	13	52	5	1		0		
Ulrich_ PNoA-Tr	1989	271		77	17	48	2	1		0		
Unertl	1987	272		100	23	20	5	0				
Unertl_PGA	1987	272		100	18	19	1	0				
Verwaest	1997	273		100	19	185	9	0	3	4	7[†]	2
Verwaest OfA-Of	1997	273		100	17	193	6	1	7	0	3[†]	1
Verwaest PTA-Ctx	1997	273		100	22	200	9	0	10	0	13[†]	7
Wiener	1995	274		100	11	31	4	0		2		
Wiener_ PGNy	1995	274		100	11	30	1	0		0		
Winter	1992	235		92	8	92	1	0		1		1
Winter_ PTA-Ctz	1992	235		92	6	91	0	0		0		0

Table S4: Footnotes

Notes; T = Data originating from a study for which the majority of ICU admission were for trauma; C = cardio-thoracic ICU; I = Infection control intervention to entire ICU; crf = group wide candidemia risk factor

MV = percentage of group receiving mechanical ventilation; NS – Not stated; LOS is mean or median length of ICU stay; The ICU-LOS is the ICU length of stay. This is based on surrogate measures including mean (or median) length of MV were taken if the length of ICU LOS was not available in order to generate broad categories of ICU stay of <5 days, 5 to 10 days and >10 days

v _sr_n is the count of *Staphylococcus aureus* VAP; and v_can_n is the count of *Candida* isolates from patients with VAP; b_sr_n is the count of *Staphylococcus aureus* bacteremia; and b_can_n is the count of Candidemia;

b_cns_n is the count of coagulative negative *Staphylococcus* bacteremia (those studies using CDC criteria are indicate by †) and b_ent_n is the count of *Enterococcal* bacteremia.

The control group in one study by Stoutenbeek [1987] appears also as the control group in another study by this Author [1984] and is used only once in the analysis here.

TAP intervention regimens abbrevations; PTA (=P, topical polymyxin; T, topical tobramycin; A, topical amphotericin); PTA-Ctx (=P, topical polymyxin; T, topical tobramycin; A, topical amphotericin; Ctx, parenteral cephalosporin); P (P = polymyxin either aerosolized or topical); PNeV (P = polymyxin; Ne = Neomycin; V = Vancomycin); PGA-Ctx (=P, topical polymyxin; G, topical gentamicin; A, topical amphotericin; Ctx, parenteral cephalosporin); PTAM (=P, topical polymyxin; T, topical tobramycin; A, topical amphotericin; topical mupirocin); E (=E, topical erythromycin); PNoA-Ctx (=P, topical polymyxin; No, topical norfloxacin; A, topical amphotericin; Ctx, parenteral cephalsporin); PGV (=P, topical polymyxin; G, topical gentamicin; V, topical vancomycin); PGNy-Ctx (=P, topical polymyxin; G, topical gentamicin; Ny, topical nystatin; Ctx, parenteral cephalsporin); P-Ctx (=P, topical polymyxin; Ctx, parenteral cephalosporin); PTAV (=P, topical polymyxin; T, topical tobramycin; A, topical amphotericin; V, topical vancomycin); PGA (=P, topical polymyxin; G, topical gentamicin; A, topical amphotericin); PTNeA (=P, topical polymyxin; T, topical tobramycin; Ne, topical Neomycin; A, topical amphotericin); PGNy (=P, topical polymyxin; G, topical gentamicin; Ny, topical nystatin); PTA-Cz (=P, topical polymyxin; T, topical tobramycin; A, topical amphotericin; Cz, parenteral Ceftazidime).

Table S5: Groups of studies of antifungal prophylaxis

Author & regimen	Year	Ref	Notes	MV	LOS	Patients	RT Candida	Candidiemia
				%	d	n	v_can_n	b_can_n
Ables	2000	275	crf	95	8	60		0
Ables_Fluc	2000	275	crf	95	8	59		0
Eggimann	1999	276	crf	NS	NS	20		2
Eggimann_Fluc	1999	276	crf	NS	NS	23		0
Giglio	2012	277		100	15	50	0	0
Giglio_Ny	2012	277		100	15	49	0	0
Jacobs	2003	278		NS	11	39		1
Jacobs_Fluc	2003	278		NS	11	32		0
Lumbreras	1996	279	crf	NS	28	67		0
Lumbreras_Fluc	1996	279	crf	NS	28	76		1
Normand	2005	280		100	12	47	0	0
Normand_Ny	2005	280		100	12	51	0	0
Ostrosky-Zeichner	2014	281	crf	100	7	102		7
Ostrosky-Zeichner_ Casp	2014	281		100	7	117		1
Savino	1994	282		31	16	72		0
Savino _Cl_ket_ny	1994	282		23	8	80		0
Savino	1994	282	crf	33	16	65		2
Savino _Cl_ket_ny	1994	282	crf	39	15	75		7
Schuster	2008	283		NS	12	122		2
Schuster_Fluc	2008	283		NS	12	122		0

Table S5: Footnotes

Notes; T = Data originating from a study for which the majority of ICU admission were for trauma; C = cardio-thoracic ICU; I = Infection control intervention to entire ICU; crf = group wide candidemia risk factor

MV = percentage of group receiving mechanical ventilation; NS – Not stated; LOS is mean or median length of ICU stay; The ICU-LOS is the ICU length of stay. This is based on surrogate measures including mean (or median) length of MV were taken if the length of ICU LOS was not available in order to generate broad categories of ICU stay of <5 days, 5 to 10 days and >10 days

RT Candida is respiratory tract candida. v_can_n is the count of *Candida* isolates from patients with VAP; b_can_n is the count of Candidemia

Fluc = fluconazole; Ny = Nystatin; Casp = Caspofungin; Cl_Ket_Ny = clotrimazole/Ketoconazole/Nystatin.

References

S1. A'Court CH, Garrard CS, Crook D, Bowler I, Conlon C, Peto T, Anderson E: Microbiological lung surveillance in mechanically ventilated patients, using non-directed bronchial lavage and quantitative culture. Q J Med. 1993;86:635-48.

S2. Alvarez-Lerma F, ICU-acquired Pneumonia Study Group. Modification of empiric antibiotic treatment in patients with pneumonia acquired in the intensive care unit. *Intens Care Med.* 1996;22(5):387-94.

S3. Antonelli M, Moro ML, Capelli O, De Blasi RA, D'Errico RR, Conti G, Bufi M, Gasparetto A: Risk factors for early onset pneumonia in trauma patients. Chest. 1994;105:224-228

S4. Apostolopoulou E, Bakakos P, Katostaras T, Gregorakos L: Incidence and risk factors for ventilator-associated pneumonia in 4 multidisciplinary intensive care units in Athens, Greece. Respir Care. 2003;48: 681-688.

S5. Arumugam SK, Mudali I, Strandvik G, El-Menyar A, Al-Hassani A, Al-Thani H. Risk factors for ventilator-associated pneumonia in trauma patients: A descriptive analysis. World J Emerg Med. 2018;9(3):203.

S6. Azoulay E, Timsit JF, Tafflet M, de Lassence A, Darmon M, Zahar JR, Schlemmer B. *Candida* colonization of the respiratory tract and subsequent pseudomonas ventilator-associated pneumonia. *Chest* 2006;129(1):110-117.

S7. Bailly S, Bouadma L, Azoulay E, Orgeas MG, Adrie C, Souweine B, Schwebel C, Maubon D, Hamidfar-Roy R, Darmon M, Wolff M. Failure of empirical systemic antifungal therapy in mechanically ventilated critically ill patients. *Am J Respir Crit Care Med.* 2015;191(10):1139-46.

S8. Baldesi O, Bailly S, Ruckly S, Lepape A, L'Heriteau F, Aupee M, Boussat S, Bervas C, Machut A, Berger-Carbonne A, Savey A. ICU-acquired candidaemia in France: epidemiology and temporal trends, 2004–2013–a study from the REA-RAISIN network. J Infect. 2017;75(1):59-67.

S9. Bekaert M, Timsit JF, Vansteelandt S, Depuydt P, Vésin A, Garrouste-Orgeas M, Decruyenaere J, Clec'h C, Azoulay E, Benoit D. Attributable mortality of ventilator-associated pneumonia: a reappraisal using causal analysis. *Am J Respir Crit Care Med*. 2011;184(10):1133-9.

S10. Bercault N, Boulain T: Mortality rate attributable to ventilator-associated nosocomial pneumonia in an adult intensive care unit: a prospective case-control study. Crit Care Med. 2001;29:2303-2309

S11. Bercault N, Wolf M, et al. Intrahospital transport of critically ill ventilated patients: a risk factor for ventilator-associated pneumonia--a matched cohort study. *Crit Care Med* 2005;33:2471-8.

S12. Berrouane Y, Daudenthun I, Riegel B, Emery MN, Martin G, Krivosic R, Grandbastien B. Early onset pneumonia in neurosurgical intensive care unit patients. J Hosp Infect. 1998;40(4):275-80.

S13. Bloos F, Held J, Kluge S, Simon P, Kogelmann K, de Heer G, Kuhn SO, Jarczak D, Motsch J, Hempel G, Weiler N. (1→ 3)-β-d-Glucan-guided antifungal therapy in adults with sepsis: the CandiSep randomized clinical trial. *Intensive Care Med.* 2022;48(7):865-75.

S14. Blot S, Koulenti D, Dimopoulos G, Martin C, Komnos A, Krueger WA, Spina G, Armaganidis A, Rello J. Prevalence, risk factors, and mortality for ventilator-associated pneumonia in middle-aged, old, and very old critically ill patients. Crit Care Med. 2014;42(3):601-9.

S15. Bochicchio GV, Joshi M, Bochicchio K, Tracy K, Scalea TM: A time-dependent analysis of intensive care unit pneumonia in trauma patients. J Trauma. 2004;56:296-301.

S16. Bonten MJ, Gaillard CA, van Tiel FH, Smeets HG, van der Geest S, Stobberingh EE: The stomach is not a source for colonization of the upper respiratory tract and pneumonia in ICU patients. Chest. 1994;105(3):878-84.

S17. Boots RJ, Phillips GE, George N, Faoagali JL. Surveillance culture utility and safety using low-volume blind bronchoalveolar lavage in the diagnosis of ventilator-associated pneumonia. Respirology. 2008;13:87-96.

S18. Bornstain C, Azoulay E, De Lassence A, Cohen Y, Costa MA, Mourvillier B, Descorps-Declere A, Garrouste-Orgeas M, Thuong M, Schlemmer B, Timsit JF: Sedation, sucralfate, and antibiotic use are potential means for protection against early-onset ventilator-associated pneumonia. Clin Infect Dis. 2004;38(10):1401-8.

S19. Borzotta AP, Beardsley K (1999) Candida infections in critically ill trauma patients: a retrospective case-control study. Arch Surg 134(6):657-665

S20. Braun SR, Levin AB, Clark KL. Role of corticosteroids in the development of pneumonia in mechanically ventilated head-trauma victims. Crit Care Med 1986;14:198-201

S21. Bregeon F, Papazian L, Visconti A, Gregoire R, Thirion X, Gouin F: Relationship of microbiologic diagnostic criteria to morbidity and mortality in patients with ventilator-associated pneumonia. JAMA. 1997;277: 655-662

S22. Bronchard R, Albaladejo P, Brezac G, et al. Early onset pneumonia: risk factors and consequences in head trauma patients. *Anesthesiology* 2004;100:234-9.

S23. Cade JF, McOwat E, Siganporia R, Keighley C, Presneill J, Sinickas V: Uncertain relevance of gastric colonization in the seriously ill. *Intensive Care Med.* 1992;18:210-217

S24. Cavalcanti M, Ferrer M, Ferrer R, Morforte R, Garnacho A, Torres A: Risk and prognostic factors of ventilator-associated pneumonia in trauma patients. Crit Care Med. 2006;34:1067-1072

S25. Cendrero JA, Solé-Violán J, Benitez AB, Catalán JN, Fernández JA, Santana PS, de Castro FR: Role of different routes of tracheal colonization in the development of pneumonia in patients receiving mechanical ventilation. Chest. 1999;116:462-470

S26. Chaari A, El Habib M, Ghdhoun H, Algia NB, Chtara K, Hamida CB, Chelly H, Bahloul M, Bouaziz M. Does low-dose hydrocortisone therapy prevent ventilator-associated pneumonia in trauma patients?. Am J Therap. 2015;22(1):22-8.

S27. Charles PE, Dalle F, Aube H, Doise JM, Quenot JP, Aho LS, Blettery B (2005) Candida spp. colonization significance in critically ill medical patients: a prospective study. Intensive Care Med 31(3):393-400.

S28. Chastre J, Trouillet JL, Vuagnat A, Joly-Guillou ML, Clavier H, Dombret MC, Gibert C: Nosocomial pneumonia in patients with acute respiratory distress syndrome. *Am J Respir Crit Care Med.* 1998;157:1165-1172

S29. Chevret S, Hemmer M, Carlet J: Incidence and risk factors of pneumonia acquired in intensive care units. Results from a multicenter prospective study on 996 patients. European Cooperative Group on Nosocomial Pneumonia. Intensive Care Med. 1993;19:256-264

S30. Combes P, Fauvage B, Oleyer C. Nosocomial pneumonia in mechanically ventilated patients, a prospective randomised evaluation of the Stericath closed suctioning system. *Intensive Care Med* 2000;26:878-82.

S31. Cook A, Norwood S, Berne J: Ventilator-associated pneumonia is more common and of less consequence in trauma patients compared with other critically ill patients. J Trauma Acute Care Surg. 2010;69(5):1083-91.

S32. Craven DE, Kunches LM, Lichtenberg DA, Kollisch NR, Barry MA, Heeren TC, McCabe WR: Nosocomial infection and fatality in medical and surgical intensive care unit patients. Arch Intern Med. 1988;148:1161-1168

S33. Daschner F, Kappstein I, Schuster F, Scholz R, Bauer E, Jooβens D, Just H: Influence of disposable ('Conchapak') and reusable humidifying systems on the incidence of ventilation pneumonia. J Hosp Infect. 1988;11:161-168

S34. De Waele JJ, Vogelaers D, Blot S Colardyn F (2003) Fungal infections in patients with severe acute pancreatitis and the use of prophylactic therapy. Crit Care 2003: 7(2) 1.

S35. De Latorre FJ, Pont T, Ferrer A, Rosselló J, Palomar M, Planas M: Pattern of tracheal colonization during mechanical ventilation. Am J Respir Crit Care Med. 1995;152:1028-1033

S36. De Santis V, Gresoiu M, Corona A, Wilson AP, Singer M. Bacteraemia incidence, causative organisms and resistance patterns, antibiotic strategies and outcomes in a single university hospital ICU: continuing improvement between 2000 and 2013. J Antimicrob Chemoth. 2015;70(1):273-8.

S37. El-Masri MM, Hammad TA, McLeskey SW, Joshi M, Korniewicz DM. Predictors of nosocomial bloodstream infections among critically ill adult trauma patients. *Infect Cont & Hosp Epidemiol.* 2004;25(8):656-63.

S38. Ensminger SA, Wright RS, Baddour LM, Afess B: Suspected ventilator-associated pneumonia in cardiac patients admitted to the coronary care unit. Mayo Clin Proc. 2006;81:32–35

S39. Ertugrul BM, Yildirim A, Ay P, Oncu S, Cagatay A, Cakar N, Ertekin C, Ozsut H, Eraksoy H, Calangu S. Ventilator-associated pneumonia in surgical emergency intensive care unit. Saudi Med J. 2006;27(1):52.

S40. Esteve F, Pujol M, Limon E, Saballs M, Argerich MJ, Verdaguer R, Manez R, Ariza X, Gudiol F. Bloodstream infection related to catheter connections: a prospective trial of two connection systems. *J Hosp Infect*. 2007;67(1):30-4.

S41. Evans HL, Zonies DH, Warner KJ, Bulger EM, Sharar SR, Maier RV, Cuschieri J. Timing of intubation and ventilator-associated pneumonia following injury. Arch Surg. 2010;145(11):1041-6.

S42. Ewig S, Torres A, El-Ebiary M, Fàbregas N, Hernandez C, Gonzalez J, Nicolas JM, Soto L: Bacterial colonization patterns in mechanically ventilated patients with traumatic and medical head injury. Incidence, risk factors, and association with ventilator-associated pneumonia. Am J Respir Crit Care Med. 1999;159:188-198

S43. Fabian TC, Boucher BA, Croce MA, Kuhl DA, Janning SW, Coffey BC, Kudsk KA: Pneumonia and stress ulceration in severely injured patients: a prospective evaluation of the effects of stress ulcer prophylaxis. Arch Surg. 1993;128(2):185-92.

S44. Fagon JY, Chastre J, Domart Y, Trouillet JL, Pierre J, Darne C, Gibert C: Nosocomial pneumonia in patients receiving continuous mechanical ventilation. Prospective analysis of 52 episodes with use of a protected specimen brush and quantitative culture techniques. *Am Rev Respir Dis* 1989;139:877-884.

S45. Ferreira D, Grenouillet F, Blasco G, Samain E, Hénon T, Dussaucy A, Millon L, Mercier M, Pili-Floury S. Outcomes associated with routine systemic antifungal therapy in critically ill patients with Candida colonization. Intens care Med. 2015;41(6):1077-88.

S46. Gacouin A, Barbarot N, Camus C, Salomon S, Isslame S, Marque S, Lavoué S, Donnio PY, Thomas R, Le Tulzo Y. Late-onset ventilator-associated pneumonia in nontrauma intensive care unit patients. Anesth Analg. 2009;109(5):1584-90.

S47. García-Garmendia JL, Ortiz-Leyba C, Garnacho-Montero J, Jiménez-Jiménez FJ, Pérez-Paredes C, Barrero-Almodóvar AE, Miner MG. Risk factors for Acinetobacter baumannii nosocomial bacteremia in critically ill patients: a cohort study. *Clin Infect Dis*. 2001;33(7):939-46.

S48. Garrouste-Orgeas M, Chevret S, Arlet G, Marie O, Rouveau M, Popoff N: Oropharyngeal or gastric colonization and nosocomial pneumonia in adult intensive care unit patients. A prospective study based on genomic DNA analysis. Am J Respir Crit Care Med. 1997;156(5):1647-56.

S49. Garrouste-Orgeas M, Timsit JF, Tafflet M, Misset B, Zahar JR, Soufir L, Carlet J: Excess risk of death from intensive care unit—acquired nosocomial bloodstream infections: a reappraisal. *Clin Infect Dis* 2006, 42:1118-1126.

S50. George DL, Falk PS, Wunderink RG, Leeper Jr KV, Meduri GU, Steere EL, Glen Mayhall C: Epidemiology of ventilator-acquired pneumonia based on protected bronchoscopic sampling. Am J Respir Crit Care Med. 1998;158:1839-1847

S51. Georges H, Leroy O, Guery B, Alfandari S, Beaucaire G: Predisposing factors for nosocomial pneumonia in patients receiving mechanical ventilation and requiring tracheotomy. Chest. 2000;118:767–774.

S52. Giamarellos-Bourboulis EJ, Bengmark S, Kannellakopoulou K, Kotzampassi K: Pro- and synbiotics to control inflammation and infection in patients with multiple injuries. *J Trauma* 2009;67:815-821.

S53. Giard M, Lepape A, Allaouchiche B, Guerin C, Lehot JJ, Robert MO, Vanhems P: Early-and late-onset ventilator-associated pneumonia acquired in the intensive care unit: comparison of risk factors. *J Crit Care* 2008, 23:27-33.

S54. Gruson D, Hilbert G, Vargas F, Valentino R, Bebear C, Allery A, Bebear C, Gbikpi-Benissan GE, Cardinaud JP: Rotation and restricted use of antibiotics in a medical intensive care unit: impact on the incidence of ventilator-associated pneumonia caused by antibiotic-resistant gram-negative bacteria. Am J Respir Crit Care Med. 2000, 162(3):837-43.

S55. Gruson D, Hilbert G, Vargas F, Valentino R, Bui N, Pereyre S, Bebear C, Bebear CM, Gbikpi-Benissan G: Strategy of antibiotic rotation: long-term effect on incidence and susceptibilities of Gram-negative bacilli responsible for ventilator-associated pneumonia. Crit Care Med. 2003;31:1908-1914.

S56. Guérin C, Girard R, Chemorin C, De Varax R, Fournier G: Facial mask noninvasive mechanical ventilation reduces the incidence of nosocomial pneumonia. Intens care Med. 1997;23(10):1024-32.

S57. Gursel G, Aydogdu M, Nadir Ozis T, Tasyurek S. Comparison of the value of initial and serial endotracheal aspirate surveillance cultures in predicting the causative pathogen of ventilator-associated pneumonia. Scand J Infect Dis. 2010, 42:341-346.

S58. Heyland DK, Cook DJ, Schoenfeld PS, Frietag A, Varon J, Wood G: The effect of acidified enteral feeds on gastric colonization in critically ill patients: results of a multicenter randomized trial. Canadian Critical Care Trials Group. Crit Care Med. 1999;27:2399-2406

S59. Holzapfel L, Chevret S, Madinier G, Ohen F, Demingeon G, Coupry A, Chaudet M: Influence of long-term oro- or nasotracheal intubation on nosocomial maxillary sinusitis and pneumonia: results of a prospective, randomized, clinical trial. Crit Care Med. 1993;21:1132-1138

S60. Huang SS, Septimus E, Kleinman K, Moody J, Hickok J, Avery TR, Lankiewicz J, Gombosev A, Terpstra L, Hartford F, Hayden MK. Targeted versus universal decolonization to prevent ICU infection. *N Engl J Med*. 2013;368(24):2255-65.

S61. Hugonnet S, Uçkay I, Pittet D Staffing level: a determinant of late-onset ventilator-associated pneumonia. Crit Care. 2007;11(4):R80

S62. Hyllienmark P, Gardlund B, Persson JO, Ekdahl K. Nosocomial pneumonia in the ICU: a prospective cohort study. Scand J Infect Dis. 2007;39:676-82.

S63. Hyllienmark P, Brattström O, Larsson E, Martling CR, Petersson J, Oldner A: High incidence of post-injury pneumonia in intensive care-treated trauma patients. Acta Anaesthesiologica Scandinavica. 2013;57(7):848-54.

S64. Ibáñez J, Peñafiel A, Marsé P, Jordá R, Raurich JM, Mata F: Incidence of gastroesophageal reflux and aspiration in mechanically ventilated patients using small-bore nasogastric tubes. J Parenteral and Enteral Nutrition. 2000;24(2):103-6.

S65. Ibrahim EH, Ward S, Sherman G, Kollef MH: A comparative analysis of patients with early-onset vs late-onset nosocomial pneumonia in the ICU setting. Chest. 2000;117:1434-1442

S66. Ibrahim EH, Sherman G, Ward S, Fraser VJ, Kollef MH. The influence of inadequate antimicrobial treatment of bloodstream infections on patient outcomes in the ICU setting. Chest. 2000;118(1):146-55.

S67. Ibrahim EH, Tracy L, Hill C, et al. The occurrence of ventilator-associated pneumonia in a community hospital: risk factors and clinical outcomes. *Chest* 2001;120:555-61.

S68. Jacobs S, Chang RW, Lee B, Bartlett FW: Continuous enteral feeding: a major cause of pneumonia among ventilated intensive care unit patients. *JPEN J Parenter Enteral Nutr* 1990;14:353-6.

S69. Jaillette E, Nseir S: Relationship between inhaled β2-agonists and ventilator-associated pneumonia: A cohort study. Critical Care Med. 2011;39(4):725-30.

S70. Jensen JUS, Hein L, Lundgren B, Bestle MH, Mohr T, Andersen MH, Procalcitonin And Survival Study Group (2015) Invasive *Candida* Infections and the Harm From Antibacterial Drugs in Critically Ill Patients: Data From a Randomized, Controlled Trial to Determine the Role of Ciprofloxacin, Piperacillin-Tazobactam, Meropenem, and Cefuroxime. *Crit Care Medicine* 2015;43(3):594-602.

S71. Jiménez P, Torres A, Rodríguez-Roisin R, de la Bellacasa JP, Aznar R, Gatell JM, Agustí-Vidal A: Incidence and etiology of pneumonia acquired during mechanical ventilation. Crit Care Med. 1989;17:882-5.

S72. Kautzky S, Staudinger T, Presterl E. Invasive Candida infections in patients of a medical intensive care unit. Wiener klinische Wochenschrift. 2015;127(3-4):132-42.

S73. Ko HK, Yu WK, Lien TC, Wang JH, Slutsky AS, Zhang H, Kou YR. Intensive care unit-acquired bacteremia in mechanically ventilated patients: clinical features and outcomes. *PloS one*. 2013;8(12):e83298.

S74. Kollef MH: Ventilator-associated pneumonia. A multivariate analysis. JAMA. 1993;270:1965-70.

S75. Kollef MH, Silver P, Murphy DM, Trovillion E: The effect of late-onset ventilator-associated pneumonia in determining patient mortality. Chest. 1995;108: 1655-62.

S76. Kollef MH, Shapiro SD, Fraser VJ, Silver P, Murphy DM, Trovillion E, Hearns ML, Richards RD, Cracchilo L, Hossin L: Mechanical ventilation with or without 7-day circuit changes. A randomized controlled trial. Ann Intern Med.1995;123:168–174

S77. Kollef MH, Von Harz B, Prentice D, Shapiro SD, Silver P, John RS, Trovillion E: Patient transport from intensive care increases the risk of developing ventilator-associated pneumonia. Chest. 1997;112(3):765-773.

S78. Kollef MH, Vlasnik JO, Sharpless L, Pasque C, Murphy D, Fraser V. Scheduled change of antibiotic classes: a strategy to decrease the incidence of ventilator-associated pneumonia. Am J Resp Crit Care Med. 1997;156(4):1040-8.

S79. Koss WG, Khalili TM, Lemus JF, Chelly MM, Margulies DR, Shabot MM: Nosocomial pneumonia is not prevented by protective contact isolation in the surgical intensive care unit. Am Surg. 2001;67:1140-4.

S80. Kunac A, Sifri ZC, Mohr AM, Horng H, Lavery RF, Livingston DH: Bacteremia and Ventilator-Associated Pneumonia: A Marker for Contemporaneous Extra-Pulmonic Infection. Surg Infect. 2014;15:77-83.

S81. Laggner AN, Lenz K, Base W, Druml W, Schneeweiss B, Grimm G: Prevention of upper gastrointestinal bleeding in long-term ventilated patients. Sucralfate versus ranitidine. Am J Med. 1989;86:81-4.

S82. Lambert ML, Suetens C, Savey A, Palomar M, Hiesmayr M, Morales I, Agodi A, Frank U, Mertens K, Schumacher M, Wolkewitz M. Clinical outcomes of health-care-associated infections and antimicrobial resistance in patients admitted to European intensive-care units: a cohort study. Lancet Infect Dis. 2011;11(1):30-8.

S83. Laupland KB, Zygun DA, Davies HD, Church DL, Louie TJ, Doig CJ Population-based assessment of intensive care unit-acquired bloodstream infections in adults: incidence, risk factors, and associated mortality rate. *Crit Care Med* 2002;30:2462-2467.

S84. Laupland KB, Kirkpatrick AW, Church DL, Ross T, Gregson DB Intensive-care-unit-acquired bloodstream infections in a regional critically ill population. *J Hosp Infect* 2004;58(2): 137-145.

S85. León C, Ruiz-Santana S, Saavedra P, Almirante B, Nolla-Salas J, Garnacho-Montero J, León MÁ. A bedside scoring system (“Candida score”) for early antifungal treatment in nonneutropenic critically ill patients with Candida colonization. Crit Care Med. 2006 Mar 1;34(3):730-7.

S86. León C, Ruiz-Santana S, Saavedra P, Galván B, Blanco A, Castro C, Balasini C, Utande-Vázquez A, de Molina FJ, Blasco-Navalproto MA, López MJ. Usefulness of the “Candida score” for discriminating between Candida colonization and invasive candidiasis in non-neutropenic critically ill patients: a prospective multicenter study. Crit Care Med. 2009;37(5):1624-33.

S87. León C, Ruiz-Santana S, Saavedra P, Castro C, Loza A, Zakariya I, Úbeda A, Parra M, Macías D, Tomás JI, Rezusta A. Contribution of Candida biomarkers and DNA detection for the diagnosis of invasive candidiasis in ICU patients with severe abdominal conditions. Crit Care. 2016;20(1):149.

S88. Lepelletier D, Roquilly A, Mahe PJ, Loutrel O, Champin P, Corvec S, Naux E, Pinaud M, Lejus C, Asehnoune K. Retrospective analysis of the risk factors and pathogens associated with early-onset ventilator-associated pneumonia in surgical-ICU head-trauma patients. J Neurosurg Anesthesiol. 2010;22(1):32-7.

S89. Li Z, Jiang C, Dong D, Zhang L, Tian Y, Ni Q, Mao E, Peng Y. The correlation between Candida colonization of distinct body sites and invasive candidiasis in emergency intensive care units: statistical and molecular biological analysis. Mycopathologia. 2016;181(7-8):475-84.

S90. Luna CM, Blanzaco D, Niederman MS, et al. Resolution of ventilator-associated pneumonia: prospective evaluation of the clinical pulmonary infection score as an early clinical predictor of outcome. Crit Care Med. 2003;31:676-682.

S91. Luyt CE, Guérin V, Combes A, Trouillet JL, Ayed SB, Bernard M, Gibert C, Chastre J: Procalcitonin kinetics as a prognostic marker of ventilator-associated pneumonia. Am J Respir Crit Care Med. 2005;171:48-53.

S92. Magnason S, Kristinsson KG, Stefansson T, Erlendsdottir H, Jonsdottir K, Kristjansson M, Gudmundsson S: Risk factors and outcome in ICU-acquired infections. Acta Anaesthesiol Scand. 2008;52:1238-1245

S93. Mahul P, Auboyer C, Jospe R, Ros A, Guerin C, el Khouri Z, Galliez M, Dumont A, Gaudin O: Prevention of nosocomial pneumonia in intubated patients respective role of mechanical subglottic secretions drainage and stress ulcer prophylaxis. Intensive Care Med. 1992;18:20-25

S94. Makris D, Manoulakas E, Komnos A, Papakrivou E, Tzovaras N, Hovas A, Zintzaras E, Zakynthinos E. Effect of pravastatin on the frequency of ventilator-associated pneumonia and on intensive care unit mortality: open-label, randomized study. Crit care med. 2011;39(11):2440-6.

S95. Markowicz P, Wolff M, Djedaini K, Cohen Y, Chastre J, Delclaux C: Multicenter prospective study of ventilator-associated pneumonia during acute respiratory distress syndrome. Incidence, prognosis, and risk factors. ARDS Study Group. Am J Respir Crit Care Med. 2000;161:1942-8.

S96. Massart N, Wattecamps G, Moriconi M, Fillatre P. Attributable mortality of ICU acquired bloodstream infections: a propensity-score matched analysis. *Eur J Clin Microbiol & Infect Dis.* 2021;40(8):1673-80.

S97. Memish ZA, Cunningham G, Oni GA, et al. The incidence and risk factors of ventilator-associated pneumonia in a Riyadh hospital. Infect Control Hosp Epidemiol. 2000;21:271-3.

S98. Michel F, Franceschini B, Berger P, Arnal JM, Gainnier M, Sainty JM, Papazian L. Early antibiotic treatment for BAL-confirmed ventilator-associated pneumonia: a role for routine endotracheal aspirate cultures. Chest. 2005;127(2):589-97.

S99. Mitsogianni M, Vasileiadis I, Parisi M, Tzanis G, Kampisiouli E, Psaroudaki Z, Perivolioti E, Fountoulis K, Routsi C, Nanas S, Tsiodras S. A Multifaceted Intervention Program to Prevent Bloodstream Infection in an IntensiveCare Unit. *Health Science J*. 2016;10(2):1.

S100. Moine P, Timsit JF, De Lassence A, Troché G, Fosse JP, Alberti C, Cohen Y: Mortality associated with late-onset pneumonia in the intensive care unit: results of a multi-center cohort study. Intensive Care Med. 2002;28:154-163

S101. Montecalvo MA, Steger KA, Farber HW: Nutritional outcome and pneumonia in critical care patients randomized to gastric versus jejunal tube feedings. The Critical Care Research Team. *Crit Care Med* 1992, 20:1377-1387.

S102. Myny D, Depuydt P, Colardyn F, Blot S: Ventilator-associated pneumonia in a tertiary care ICU analysis of risk factors for acquisition and mortality. Acta Clin Belg. 2005;60:114-121.

S103. Nguile-Makao M, Zahar JR, Français A, Tabah A, Garrouste-Orgeas M, Allaouchiche B, Goldgran-Toledano D, Azoulay E, Adrie C, Jamali S, Clec'h C. Attributable mortality of ventilator-associated pneumonia: respective impact of main characteristics at ICU admission and VAP onset using conditional logistic regression and multi-state models. Intens care med. 2010;36(5):781-9.

S104. Nielsen SL, Røder B, Magnussen P, Engquist A, Frimodt-møller N. Nosocomial pneumonia in an intensive care unit in a Danish university hospital: incidence, mortality and etiology. Scand J Infect Dis. 1992;24:65-70.

S105. Nseir S, Di Pompeo C, Soubrier S, Cavestri B, Jozefowicz E, Saulnier F, Durocher A: Impact of ventilator-associated pneumonia on outcome in patients with COPD. Chest. 2005;128(3):1650-1656.

S106. Nseir S, Jozefowicz E, Cavestri B, Sendid B, Di Pompeo C, Dewavrin F, Durocher A. Impact of antifungal treatment on Candida–Pseudomonas interaction: a preliminary retrospective case–control study. *Intensive Care Med* 2007;33(1):137-142.

S107. Orsi GB, Giuliano S, Franchi C, Ciorba V, Protano C, Giordano A, Rocco M, Venditti M. Changed epidemiology of ICU acquired bloodstream infections over 12 years in an Italian teaching hospital. *Minerva Anestesiol.* 2015;81(9):980-8.

S108. Osmon S, Warren D, Seiler SM, Shannon W, Fraser VJ, Kollef MH: The influence of infection on hospital mortality for patients requiring >48 h of intensive care. *Chest* 2003, 124:1021-1029.

S109. Saied WI, Mourvillier B, Cohen Y, Ruckly S, Reignier J, Marcotte G, Siami S, Bouadma L, et al on behalf of the OUTCOMEREA study group. A comparison of the mortality risk associated with ventilator-acquired bacterial pneumonia and nonventilator ICU-acquired bacterial pneumonia. Crit care med. 2019;47:345-52.

S110. Papazian L, Bregeon F, Thirion X, Gregoire R, Saux P, Denis JP, Perin G, Charrel J, Dumon JF, Affray JP, Gouin F: Effect of ventilator-associated pneumonia on mortality and morbidity. Am J Respir Crit Care Med. 1996;154:91-7.

S111. Petri MG, König J, Moecke HP, Gramm HJ, Barkow H, Kujath P, Dennhart R, Lode H. Epidemiology of invasive mycosis in ICU patients: a prospective multicenter study in 435 non-neutropenic patients. *Intensive Care Med* 1997;23(3):317-325.

S112. Potgieter PD, Linton DM, Oliver S, Forder AA: Nosocomial infections in a respiratory intensive care unit. Crit Care Med. 1987;15:495-498

S113. Prowle JR, Echeverri JE, Ligabo EV, Sherry N, Taori GC, Crozier TM, Bellomo R. Acquired bloodstream infection in the intensive care unit: incidence and attributable mortality. Crit Care 2011;15(2):R100.

S114. Ramirez P, Lopez-Ferraz C, Gordon M, Gimeno A, Villarreal E, Ruiz J, Menendez R, Torres A. From starting mechanical ventilation to ventilator-associated pneumonia, choosing the right moment to start antibiotic treatment. *Crit Care* 2016;20(1):169.

S115. Rello J, Quintana E, Ausina V, Castella J, Luquin M, Net A, Prats G: Incidence, etiology, and outcome of nosocomial pneumonia in mechanically ventilated patients. Chest. 1991;100:439-444

S116. Rello J, Ausina V, Castella J, et al Nosocomial respiratory tract infections in multiple trauma patients. Influence of level of consciousness with implications for therapy. Chest 1992;102:525-529

S117. Rello J, Ricart M, Mirelis B, Quintana E, Gurgui M, Net A, Prats, G: Nosocomial bacteremia in a medical-surgical intensive care unit: epidemiologic characteristics and factors influencing mortality in 111 episodes. *Intensive Care Med* 1994;20:94-98.

S118. Rello J, Sonora R, Jubert P, et al. Pneumonia in intubated patients: role of respiratory airway Care *Am J Respir Crit Care Med* 1996;154:111-5.

S119. Rello J, Ollendorf DA, Oster G, et al. Epidemiology and outcomes of ventilator-associated pneumonia in a large US database. Chest 2002;122:2115-2121

S120. Rello J, Lorente C, Diaz E, et al. Incidence, etiology, and outcome of nosocomial pneumonia in ICU patients requiring percutaneous tracheotomy for mechanical ventilation. *Chest*. 2003;124:2239-43.

S121. Resende MM, Monteiro SG, Callegari B, Figueiredo PM, Monteiro CR, Monteiro-Neto V. Epidemiology and outcomes of ventilator-associated pneumonia in northern Brazil: an analytical descriptive prospective cohort study. BMC infect Dis 2013;13(1): 119

S122. Reusser P, Zimmerli W, Scheidegger D, Marbet GA, Buser M, Gyr K: Role of gastric colonization in nosocomial infections and endotoxemia: a prospective study in neurosurgical patients on mechanical ventilation. J Infect Dis. 1989;160:414-421

S123. Rincón-Ferrari MD, Flores-Cordero JM, Leal-Noval SR, Murillo-Cabezas F, Cayuelas A, Muñoz-Sánchez MA, Sánchez-Olmedo JI: Impact of ventilator-associated pneumonia in patients with severe head injury. J Trauma Acute Care Surg. 2004;57(6):1234-40.

S124. Rodrigues PM, Neto C, Santos LR, Knibel MF. Ventilator-associated pneumonia: epidemiology and impact on the clinical evolution of ICU patients. Jornal brasileiro de pneumologia. 2009 Nov;35(11):1084-91.

S125. Rodriguez JL, Gibbons KJ, Bitzer LG, Dechert RE, Steinberg SM, Flint LM: Pneumonia: incidence, risk factors, and outcome in injured patients. J Trauma. 1991;31: 907-12.

S126. Ruiz-Santana S, Garcia Jimenez A, Esteban A, et al. ICU pneumonias: a multi-institutional study. *Crit Care Med*. 1987;15:930-932.

S127. Salata RA, Lederman MM, Shlaes DM, Jacobs MR, Eckstein E, Tweardy D, Toossi Z, Chmielewski R, Marino J, King CH: Diagnosis of nosocomial pneumonia in intubated, intensive care unit patients. Am Rev Respir Dis. 1987;135:426-432

S128. Shahin J, Bielinski M, Guichon C, Flemming C, Kristof AS Suspected ventilator-associated respiratory infection in severely ill patients: a prospective observational study. Crit Care 2013;17: R251

S129. Sofianou DC, Constandinidis TC, Yannacou M, Anastasiou H, Sofianos E: Analysis of risk factors for ventilator-associated pneumonia in a multidisciplinary intensive care unit. *Eur J Clin Microbiol Infect Dis* 2000, 19:460-463.

S130. Stéphan F, Mabrouk N, Decailliot F, Delclaux C, Legrand P: Ventilator-associated pneumonia leading to acute lung injury after trauma: importance of *Haemophilus influenzae*. Anesthesiology. 2006;104: 235-41.

S131. Stoclin A, Rotolo F, Hicheri Y, Mons M, Chachaty E, Gachot B, Pignon JP, Wartelle M, Blot F. Ventilator-associated pneumonia and bloodstream infections in intensive care unit cancer patients: a retrospective 12-year study on 3388 prospectively monitored patients. Supportive Care Cancer. 2020;28(1):193-200.

S132. Tan X, Zhu S, Yan D, Chen W, Chen R, Zou J, Yan J, Zhang X, Farmakiotis D, Mylonakis E. Candida spp. airway colonization: A potential risk factor for Acinetobacter baumannii ventilator-associated pneumonia. Med Mycol. 2016:myw009.

S133. Tejada Artigas AT, Dronda SB, Vallés EC, Marco JM, Usón MC, Figueras P, Suarez FJ, Hernandez A: Risk factors for nosocomial pneumonia in critically ill trauma patients. Crit Care Med. 2001;29:304-9.

S134. Thompson DS. Estimates of the rate of acquisition of bacteraemia and associated excess mortality in a general intensive care unit: a 10 year study. *J Hosp Infect*. 2008;69(1):56-61.

S135. Timsit JF, Chevret S, Valcke J, Misset B, Renaud B, Goldstein FW, Vaury P, Carlet J: Mortality of nosocomial pneumonia in ventilated patients: influence of diagnostic tools. Am J Respir Crit Care Med. 1996;154:116-23.

S136. Torres A, Aznar R, Gatell JM, Jiménez P, González J, Ferrer A, Celis R, Rodriguez-Roisin R: Incidence, risk, and prognosis factors of nosocomial pneumonia in mechanically ventilated patients. Am Rev Respir Dis. 1990;142:523-8.

S137. Trouillet JL, Chastre J, Vuagnat A, Joly-Guillou ML, Combaux D, Dombret MC, Gibert C: Ventilator-associated pneumonia caused by potentially drug-resistant bacteria. Am J Respir Crit Care Med. 1998;157(2):531-9.

S138. Urli T, Perone G, Acquarolo A, Zappa S, Antonini B, Ciani A: Surveillance of infections acquired in intensive care: usefulness in clinical practice. *J Hosp Infect* 2002, 52:130-5.

S139. Valles J, Pobo A, Garcia-Esquirol O, Mariscal D, Real J, Fernández R. Excess ICU mortality attributable to ventilator-associated pneumonia: the role of early vs late onset. *Intensive care medicine*, 2007;33(8):1363-1368.

S140. Vanhems P, Bénet T, Voirin N, Januel JM, Lepape A, Allaouchiche B, Argaud L, Chassard D, Guérin C. Early-onset ventilator-associated pneumonia incidence in intensive care units: a surveillance-based study. BMC Infect Dis. 2011;11(1):236.

S141. Verhamme KM, De Coster W, De Roo L, De Beenhouwer H, Nollet G, Verbeke J, Demeyer I, Jordens P: Pathogens in early-onset and late-onset intensive care unit–acquired pneumonia. Infection Control Hospital Epidemiol. 2007;28(4):389-397.

S142. Violan JS, Sanchez-Ramirez C, Mujica AP, Cendrero JC, Fernandez JA, de Castro FR: Impact of nosocomial pneumonia on the outcome of mechanically-ventilated patients. Crit Care (Lond). 1998;2:19-23.

S143. Warren DK, Zack JE, Elward AM, Cox MJ, Fraser VJ. Nosocomial primary bloodstream infections in intensive care unit patients in a nonteaching community medical center: a 21-month prospective study. *Clin Infect Dis.* 2001;33(8):1329-35.

S144. Woske HJ, Röding T, Schulz I, Lode H: Ventilator-associated pneumonia in a surgical intensive care unit Epidemiology, etiology and comparison of three bronchoscopic methods for microbiological specimen sampling. Crit Care. 2001;5:167–173.

S145. Xie DS, Xiong W, Lai RP, Liu L, Gan XM, Wang XH, Nie SF. Ventilator-associated pneumonia in intensive care units in Hubei Province, China: a multicentre prospective cohort survey. J Hosp Infect 2011;78(4): 284-288

S146. Zahar JR, Nguile-Makao M, Français A, Schwebel C, Garrouste-Orgeas M, Goldgran-Toledano D, Azoulay E, Thuong M, Jamali S, Cohen Y, De Lassence A. Predicting the risk of documented

ventilator-associated pneumonia for benchmarking: construction and validation of a score. Crit care med. 2009;37(9):2545-51.

S147. Acosta-Escribano J, Fernández-Vivas M, Carmona TG, Caturla-Such J, Garcia-Martinez M, Menendez-Mainer A, Sanchez-Payá J (2010) Gastric versus transpyloric feeding in severe traumatic brain injury: a prospective, randomized trial. Intensive Care Med 36:1532-1539

S148. Bonten MJ, Gaillard CA, Van der Geest S, Van Tiel FH, Beysens AJ, Smeets HG, Stobberingh EE: The role of intragastric acidity and stress ulcer prophylaxis on colonization and infection in mechanically ventilated ICU patients. A stratified, randomized, double-blind study of sucralfate versus antacids. Am J Respir Crit Care Med. 1995;152:1825-1834.

S149. Cook D, Guyatt G, Marshall J, et al A comparison of sucralfate and ranitidine for the prevention of upper gastrointestinal bleeding in patients requiring mechanical ventilation. Canadian Critical Care Trials Group. N Engl J Med 1998;338:791-797

S150. Daumal F, Colpart E, Manoury B, Mariani M, Daumal M. Changing heat and moisture exchangers every 48 hours does not increase the incidence of nosocomial pneumonia. Infection Control & Hospital Epidemiology. 1999;20(5):347-9.

S151. Djedaini K, Billiard M, Mier L, Le Bourdelles G, Brun P, Markowicz P, Estagnasie P, Coste F, Boussougant Y, Dreyfuss D: Changing heat and moisture exchangers every 48 hours rather than 24 hours does not affect their efficacy and the incidence of nosocomial pneumonia. Am J Respir Crit Care Med. 1995;152(5):1562-9.

S152. Drakulovic MB, Torres A, Bauer TT, Nicolas JM, Nogué S, Ferrer M: Supine body position as a risk factor for nosocomial pneumonia in mechanically ventilated patients: a randomised trial. Lancet. 1999;354(9193):1851-1858

S153. Dreyfuss D, Djedaini K, Weber P, Brun P, Lanore JJ, Rahmani J, Coste F: Prospective study of nosocomial pneumonia and of patient and circuit colonization during mechanical ventilation with circuit changes every 48 hours versus no change. Am Rev Respir Dis. 1991;143(4 Pt 1), 738-743.

S154. Dreyfuss D, Djedaïni K, Gros I, Mier L, Le Bourdellés G, Cohen Y, Estagnasié P, Coste F, Boussougant Y: Mechanical ventilation with heated humidifiers or heat and moisture exchangers: effects on patient colonization and incidence of nosocomial pneumonia. Am J Respir Crit Care Med. 1995;151:986-92.

S155. Driks MR, Craven DE, Celli BR, et al (1987) Nosocomial pneumonia in intubated patients given sucralfate as compared with antacids or histamine type 2 blockers. The role of gastric colonization. N Engl J Med 317:1376-1382

S156. Forestier C, Guelon D, Cluytens V, Guillart T, Sirot J, De champs C: Oral probiotic and prevention of *Pseudomonas aeruginosa* infections: a randomized, double-blind, placebo controlled pilot study in intensive care unit patients. *Crit Care* 2008;12:R69.

S157. François B, Jafri HS, Chastre J, Sánchez-García M, Eggimann P, et al. Efficacy and safety of suvratoxumab for prevention of Staphylococcus aureus ventilator-associated pneumonia (SAATELLITE): a multicentre, randomised, double-blind, placebo-controlled, parallel-group, phase 2 pilot trial. The Lancet Infect Dis. 2021;21(9):1313-23.

S158. Heyland DK, Cook DJ, Schoenfeld PS, Frietag A, Varon J, Wood G The effect of acidified enteral feeds on gastric colonization in critically ill patients: results of a multicenter randomized trial. Canadian Critical Care Trials Group. Crit Care Med 1999;27:2399-2406

S159. Holzapfel L, Chastang C, Demingeon G, Bohe J, Piralla B, Coupry A: A randomized study assessing the systematic search for maxillary sinusitis in nasotracheally mechanically ventilated patients. Influence of nosocomial maxillary sinusitis on the occurrence of ventilator-associated pneumonia. Am J Respir Crit Care Med. 1999;159:695-701

S160. Kappstein I, Schulgen G, Friedrich T, Hellinger P, Benzing A, Geiger K, Daschner FD. Incidence of pneumonia in mechanically ventilated patients treated with sucralfate or cimetidine as prophylaxis for stress bleeding: bacterial colonization of the stomach. Am J Med. 1991;91(2):s125-31

S161. Kirschenbaum L, Azzi E, Sfeir T, et al. Effect of continuous lateral rotational therapy on the prevalence of ventilator-associated pneumonia in patients requiring long-term ventilatory care *Crit Care Med* 2002;30:1983-6.

S162. Kirton OC, DeHaven B, Morgan J, et al. A prospective, randomized comparison of an in-line heat moisture exchange filter and heated wire humidifiers: rates of ventilator-associated early-onset (community-acquired) or late-onset (hospital-acquired) pneumonia and incidence of endotracheal tube occlusion. *Chest* 1997;112:1055-9.

S163. Knight DJ, Gardiner D, Banks A, Snape SE, Weston VC, Bengmark S, Girling KJ: Effect of synbiotic therapy on the incidence of ventilator associated pneumonia in critically ill patients: a randomised, double-blind, placebo-controlled trial. Intensive Care Med. 2009;35:854-861.

S164. Kollef MH, Afessa B, Anzueto A, Veremakis C, Kerr KM, Margolis BD, Schinner R: Silver-coated endotracheal tubes and incidence of ventilator-associated pneumonia: the NASCENT randomized trial. JAMA. 2008;300(7):805-813

S165. Lacherade JC, Auburtin M, Cerf C, Van de Louw A, Soufir L, Rebufat Y, Rezaiguia S, Ricard JD, Lellouche F, Brun-Buisson C, Brochard L: Impact of humidification systems on ventilator-associated pneumonia: a randomized multicenter trial. Am J Respir Crit Care Med. 2005;172:1276-1282

S166. Lacherade JC, De Jonghe B, Guezennec P, Debbat K, Hayon J, Monsel A, Bastuji-Garin S: Intermittent subglottic secretion drainage and ventilator-associated pneumonia A multicenter trial. Am J Respir Crit Care Med. 2010;182:910-917.

S167. Launey Y, Nesseler N, Le Cousin A, Feuillet F, Garlantezec R, Mallédant Y, Seguin P: Effect of a fever control protocol-based strategy on ventilator-associated pneumonia in severely brain-injured patients. Crit Care. 2014;18(6):1.

S168. Lorente L, Lecuona M, Málaga J, Revert C, Mora ML, Sierra A: Bacterial filters in respiratory circuits: an unnecessary cost? Crit Care Med 2003;31:2126-2130

S169. Lorente L, Lecuona M, Galván R, Ramos MJ, Mora ML, Sierra A: Periodically changing ventilator circuits is not necessary to prevent ventilator-associated pneumonia when a heat and moisture exchanger is used. Infect Control Hosp Epidemiol. 2004;25:1077-1082

S170. Lorente L, Lecuona M, Martín MM, García C, Mora ML, Sierra A: Ventilator-associated pneumonia using a closed versus an open tracheal suction system. Crit Care Med. 2005;33:115-119

S171. Lorente L, Lecuona M, Jiménez A, Mora ML, Sierra A: Tracheal suction by closed system without daily change versus open system. Intensive Care Med. 2006;32:538-44.

S172. Lorente L, Lecuona M, Jimenez A, Mora ML, Sierra A: Ventilator-associated pneumonia using a heated humidifier or a heat and moisture exchanger: a randomized controlled trial [ISRCTN88724583]. Crit Care 2006;10:R116

S173. Lorente L, Lecuona M, Jimenez A, Mora ML, Sierra: Influence of an endotracheal tube with polyurethane cuff and subglottic secretion drainage on pneumonia. Am J Respir Crit Care Med. 2007;176:1079-1083

S174. Lorente L, Lecuona M, Jiménez A, Lorenzo L, Roca I, Cabrera J, Llanos C, Mora ML: Continuous endotracheal tube cuff pressure control system protects against ventilator-associated pneumonia. Crit Care. 2014;18(2):1.

S175. Manzano F, Fernandez-Mondejar E, Colmenero M, Poyatos ME, Rivera R, Machado J, Catalan I, Artigas A: Positive-end expiratory pressure reduces incidence of ventilator-associated pneumonia in nonhypoxemic patients. Crit Care Med: 2008;36(8):2225-31.

S176. Martin C, Perrin G, Gevaudan MJ, Saux P, Gouin F. Heat and moisture exchangers and vaporizing humidifiers in the intensive care unit. Chest. 1990;97(1):144-9.

S177. Morrow LE, Kollef MH, Casale TB: Probiotic prophylaxis of ventilator-associated pneumonia: a blinded, randomized, controlled trial. Am J Respir Crit Care Med. 2010;182:1058-1064

S178. Nseir S, Zerimech F, Fournier C, Lubret R, Ramon P, Durocher A, Balduyck M: Continuous control of tracheal cuff pressure and microaspiration of gastric contents in critically ill patients. Am J Respir Crit Care Med. 2011;184(9):1041-7.

S179. Pickworth KK, Falcone RE, Hoogeboom JE, et al Occurrence of nosocomial pneumonia in mechanically ventilated trauma patients: a comparison of sucralfate and ranitidine. Crit Care Med 1993;21:1856-1862

S180. Pneumatikos I, Konstantonis D, Tsagaris I, Theodorou V, Vretzakis G, Danielides V, Bouros D: Prevention of nosocomial maxillary sinusitis in the ICU: the effects of topically applied alpha-adrenergic agonists and corticosteroids. Intensive Care Med. 2006;32:532-537

S181. Prod'hom G, Leuenberger P, Koerfer J, Blum A, Chiolero R, Schaller MD, Perret C, Spinnler O, Blondel J, Siegrist H, Saghafi L: Nosocomial pneumonia in mechanically ventilated patients receiving antacid, ranitidine, or sucralfate as prophylaxis for stress ulcer. A randomized controlled trial. Ann Intern Med. 1994;120:653-62.

S182. Reignier J, Mercier E, Le Gouge A, Boulain T, Desachy A, Bellec F, Lascarrou JB: Effect of Not Monitoring Residual Gastric Volume on Risk of Ventilator-Associated Pneumonia in Adults Receiving Mechanical Ventilation and Early Enteral Feeding. A Randomized Controlled Trial. *JAMA* 2013, 309;249-256.

S183. Rumbak MJ, Truncale T, Newton MN, Adams B, Hazard P. A Prospective, Randomized Study Comparing Early Versus Delayed Percutaneous Tracheostomy In Critically Ill Medical Patients Requiring Prolonged Mechanical Ventilation. Chest. 2000;118(4):97S-8S.

S184. Ryan P, Dawson J, Teres D, Celoria G, Navab F: Nosocomial pneumonia during stress ulcer prophylaxis with cimetidine and sucralfate. Arch Surg. 1993;128(12):1353-7.

S185. Smulders K, van der Hoeven H, Weers-Pothoff I, Vandenbroucke-Grauls C A randomized clinical trial of intermittent subglottic secretion drainage in patients receiving mechanical ventilation. Chest 2002;121:858-862

S186. Staudinger T, Bojic A, Holzinger U, Meyer B, Rohwer M, Mallner F, Locker GJ Continuous lateral rotation therapy to prevent ventilator-associated pneumonia Crit Care Med 2010;38(2):486-490

S187. Thomachot L, Viviand X, Arnaud S, Boisson C, Martin CD: Comparing two heat and moisture exchangers, one hydrophobic and one hygroscopic, on humidifying efficacy and the rate of nosocomial pneumonia. Chest. 1998;114:1383-1389

S188. Thomachot L, Leone M, Razzouk K, Antonini F, Vialet R, Martin C: Do the components of heat and moisture exchanger filters affect humidifying efficacy and the incidence of nosocomial pneumonia? Crit Care Med. 1999;27:923–928

S189. Thomachot L, Leone M, Razzouk K, Antonini F, Vialet R, Martin C: Randomized Clinical Trial of Extended Use of a Hydrophobic Condenser Humidifier: 1 vs 7 Days. Crit Care Med. 2002;30:232-7

S190. Valencia M, Ferrer M, Farre R, Navajas D, Badia JR, Nicolas JM, Torres A: Automatic control of tracheal tube cuff pressure in ventilated patients in semirecumbent position: a randomized trial. Crit Care Med. 2007;35: 1543-9.

S191. Walaszek M, Gniadek A, Kolpa M, Wolak Z, Kosiarska A. The effect of subglottic secretion drainage on the incidence of ventilator associated pneumonia. *Biomed Pap Med Fac Univ Palacky Olomouc Czech Repub*. 2017;161(4):374-80.

S192. Zeng J, Wang CT, Zhang FS, Qi F, Wang SF, Ma S, Wu TJ, Tian H, Tian ZT, Zhang SL, Qu Y. Effect of probiotics on the incidence of ventilator-associated pneumonia in critically ill patients: a randomized controlled multicenter trial. Intens care med. 2016;42(6):1018-28.

S193. Bellissimo-Rodrigues WT, Menegueti MG, Gaspar GG, Nicolini EA, Auxiliadora-Martins M, Basile-Filho A, Bellissimo-Rodrigues F. Effectiveness of a Dental Care Intervention in the Prevention of Lower Respiratory Tract Nosocomial Infections among Intensive Care Patients: A Randomized Clinical Trial. Infect Control Hosp Epidemiol 2014, 35:1342-1348

S194. Bleasdale SC, Trick WE, Gonzalez IM, Lyles RD, Hayden MK, Weinstein RA. Effectiveness of chlorhexidine bathing to reduce catheter-associated bloodstream infections in medical intensive care unit patients. Arch intern med. 2007;167(19):2073-9.

S195. Ćabov T, Macan D, Husedžinović I, Škrlin-Šubić J, Bošnjak D, Šestan-Crnek S, Perić B, Kovač Z, Golubović V. The impact of oral health and 0.2% chlorhexidine oral gel on the prevalence of nosocomial infections in surgical intensive-care patients: a randomized placebo-controlled study. Einfluss von Mundgesundheit und von 0, 2% Chlorhexidin-Gel auf die Entwicklung von nosokomialen Infektionen bei Patienten auf einer chirurgischen Intensivstation. Wiener klinische Wochenschrift. 2010;122(13-14):397-404.

S196. Caruso P, Denari S, Ruiz SA, Demarzo SE, Deheinzelin D. Saline instillation before tracheal suctioning decreases the incidence of ventilator-associated pneumonia. Crit Care Med 2009, 37:32-38.

S197. Climo MW, Yokoe DS, Warren DK et al. Effect of daily chlorhexidine bathing on hospital-acquired infection. *N Engl J Med* 2013; 368: 533–542.

S198. Fourrier FE, Cau-Pottier H, Boutigny M, Roussel-Delvallez M, Jourdain, Chopin C: Effects of dental plaque antiseptic decontamination on bacterial colonization and nosocomial infections in critically ill patients. Intensive Care Med. 2000;26:1239-1247

S199. Fourrier F, Dubois D, Pronnier P, Herbecq P, Leroy O, Desmettre T, Roussel-Delvallez M: Effect of gingival and dental plaque antiseptic decontamination on nosocomial infections acquired in the intensive care unit a double-blind placebo-controlled multicenter study. Crit Care Med. 2005;33:1728-1735

S200. Koeman M, van der Ven AJ, Hak E, et al. Oral decontamination with chlorhexidine reduces the incidence of ventilator-associated pneumonia. Am J Respir Crit Care Med 2006;173:1348-1355

S201. Kollef M, Pittet D, Sanchez Garcia M, et al. A randomized double-blind trial of iseganan in prevention of ventilator-associated pneumonia. *Am J Respir Crit Care Med* 2006: 173:91-7.

S202. Lorente L, Lecuona M, Jiménez A, Palmero S, Pastor E, Lafuente N, Ramos MJ, Mora ML, Sierra A: Ventilator-associated pneumonia with or without toothbrushing a randomized controlled trial. Eur J Clin Microbiol Infect Dis. 2012;31:1-9

S203. Milstone AM, Elward A, Song X, et al. Pediatric SCRUB Trial Study Group. Daily chlorhexidine bathing to reduce bacteraemia in critically ill children: a multicentre, cluster-randomised, crossover trial. The Lancet. 2013 30;381:1099-106.

S204. Mori H, Hirasawa H, Oda S, Shiga H, Matsuda K, Nakamura M: Oral care reduces incidence of ventilator-associated pneumonia in ICU populations. Intensive Care Med, 2006, 32(2), 230-236.

S205. Noto MJ, Domenico HJ, Byrne DW, Talbot T, Rice TW, Bernard GR, Wheeler AP. Chlorhexidine bathing and health care–associated infections: a randomized clinical trial. *JAMA.* 2015;313(4):369-78.

S206. Seguin P, Tanguy M , Laviolle B, Tirel O, Malledant Y: Effect of oropharyngeal decontamination by povidone-iodine on ventilator-associated pneumonia in patients with head trauma. *Crit Care Med* 2006, 34:1514-1519.

S207. Seguin P, Laviolle B, Dahyot-Fizelier C, Dumont R, Veber B, Gergaud S, Asehnoune K, Mimoz O, Donnio PY, Bellissant E, Malledant Y. Effect of oropharyngeal povidone-iodine preventive oral care

on ventilator-associated pneumonia in severely brain-injured or cerebral hemorrhage patients: a multicenter, randomized controlled trial. Crit care med. 2014;42(1):1-8.

S208. Swan JT, Ashton CM, Bui LN, Pham VP, Shirkey BA, Blackshear JE, Bersamin JB, Pomer RM, Johnson ML, Magtoto AD, Butler MO. Effect of chlorhexidine bathing every other day on prevention of hospital-acquired infections in the surgical ICU: a single-center, randomized controlled trial. Crit Care Med. 2016;44(10):1822-32.

S209. Wittekamp BH, Plantinga NL, Cooper BS, Lopez-Contreras J, Coll P, Mancebo J, Wise MP, Morgan MP, Depuydt P, Boelens J, Dugernier T. Decontamination strategies and bloodstream infections with antibiotic-resistant microorganisms in ventilated patients: a randomized clinical trial. *JAMA*. 2018.

S210. Bergmans DC, Bonten MJ, Gaillard CA, et al Prevention of ventilator-associated pneumonia by oral decontamination: a prospective, randomized, double-blind, placebo-controlled study. Am J Respir Crit Care Med 2001;164:382-388

S211. Bonten MJ, Gaillard CA, Johanson Jr WG, Van Tiel FH, Smeets HG, Van Der Geest S, Stobberingh EE. Colonization in patients receiving and not receiving topical antimicrobial prophylaxis. *Am J Respir Crit Care Med* 1994;150(5):1332-1340.

S212. Camus C, Salomon S, Bouchigny C, Gacouin A, Lavoué S, Donnio PY, Javaudin L, Chapplain JM, Uhel F, Le Tulzo Y, Bellissant E. Short-term decline in all-cause acquired infections with the routine use of a decontamination regimen combining topical polymyxin, tobramycin, and amphotericin B with mupirocin and chlorhexidine in the ICU: a single-center experience. *Crit Care Med.* 2014;42(5):1121-30.

S213. de la Court JR, Sigaloff KC, Groot T, van der Spoel JI, Schade RP. Reducing the dosing frequency of selective digestive tract decontamination to three times daily provides effective decontamination of Gram-negative bacteria. *Eur J Clin Microbiol & Infect Dis*. 2021;40(9):1843-50.

S214. de Smet AMGA, Kluytmans JAJW, Cooper BS, Mascini EM, Benus RFJ, et al: Decontamination of the digestive tract and oropharynx in ICU patients. *N Engl J Med* 2009, 360:20–31.

S215. Garbino J, Lew DP, Romand JA, Hugonnet S, Auckenthaler R, Pittet D: Prevention of severe Candida infections in nonneutropenic, high-risk, critically ill patients: a randomized, double-blind, placebo-controlled trial in patients treated by selective digestive decontamination. Intensive Care Med 2002;28:1708-1717

S216. Godard J, Guillaume C, Reverdy ME, Bachmann P, Bui-Xuan B, Nageotte A, Motin J: Intestinal decontamination in a polyvalent ICU. A double-blind study. *Intensive Care Med* 1990, 16:307-311.

S217. Hartenauer U, Thulig B, Diemer W, Lawin P, Fegeler W, Kehrel R, Ritzerfeld W Effect of selective flora suppression on colonization, infection, and mortality in critically ill patients: a one-year, prospective consecutive study. *Crit Care Med* 1991;19:463-473

S218. Hjortrup A, Rasmussen A, Hansen BA, Hoiby N, Heslet L, Moesgaard F, Kirkegaard P (1997) Early bacterial and fungal infections in liver transplantation after oral selective bowel decontamination. *Transplantation proceedings* 29:3106-3110

S219. Konrad F, Schwalbe B, Heeg K, et al. [Frequency of colonization and pneumonia and development of resistance in long-term ventilated intensive-care patients subjected to selective decontamination of the digestive tract]. *Anaesthesist* 1989;38:99-109.

S220. Landelle C, Boyer VN, Abbas M, Genevois E, Abidi N, Naimo S, Raulais R, Bouchoud L, Boroli F, Terrisse H. Impact of a multifaceted prevention program on ventilator-associated pneumonia including selective oropharyngeal decontamination. *Intensive Care Med* 2018;44(11):1777-86.

S221. Ledingham I, Eastaway A, Mckay I, Alcock S, Mcdonald J, Ramsay G: Triple regimen of selective decontamination of the digestive tract, systemic cefotaxime, and microbiological surveillance for prevention of acquired infection in intensive care. *Lancet* 1988, 1:785-90.

S222. Leone M, Bourgoin A, Giuly E, et al. Influence on outcome of ventilator-associated pneumonia in multiple trauma patients with head trauma treated with selected digestive decontamination. *Crit Care Med* 2002; 30:1741-6.

S223. Mathieu C, Abbate R, Meresse Z, Hammad E, Duclos G, Antonini F, Cassir N, Schouten J, Zieleskiewicz L, Leone M. Decreased duration of intravenous cephalosporins in intensive care unit patients with selective digestive decontamination: a retrospective before-and-after study. *Eur J Clin Microbiol & Infect Dis*. 2020;39(11):2115-20.

S224. Nardi G, Valentinis U, Bartaletti R, Bello A, De AM, Muzzi R, Giordano F, Troncon MG. Effectiveness of topical selective decontamination, without systemic antibiotic prophylaxis, in prevention of pulmonary infection in intensive care. *Minerva anestesiologica.* 1990;56(1-2):19-26.

S225. Nardi G, Di Silvestre A, De Monte A, Massarutti D, Proietti A, Troncon MG, Zussino M: Reduction in gram-positive pneumonia and antibiotic consumption following the use of a SDD protocol including nasal and oral mupirocin. *Eur J Emerg Med* 2001;8:203-214

S226. Ong DS, Bonten MJ, Safdari K, Spitoni C, Frencken JF, Witteveen E, Horn J, Klein Klouwenberg PM, Cremer OL, MARS consortium, de Beer FM. Epidemiology, management, and risk-adjusted mortality of ICU-acquired enterococcal bacteremia. *Clin Infect Dis* 2015;61(9):1413-20.

S227. Oostdijk EAN, Kesecioglu J, Schultz MJ, et al. Notice of Retraction and Replacement: Oostdijk et al. Effects of Decontamination of the Oropharynx and Intestinal Tract on Antibiotic Resistance in ICUs: A Randomized Clinical Trial. *JAMA*. 2014;312(14):1429-1437. *JAMA* 2017

S228. Rouby JJ, Poete P, de Lassale EM, Nicolas MH, Bodin L, Jarlier V, Korinek AM, Viars P. Prevention of Gram negative noscomial bronchopneumonia by intratracheal colistin in critically ill patients. *Intensive Care Med*. 1994;20(3):187-92.

S229. Silvestri L, Bragadin CM, Milanese M, Gregori D, Consales C, Gullo A, Van Saene HK. Are most ICU infections really nosocomial? A prospective observational cohort study in mechanically ventilated patients. *J Hosp Infect.* 1999;42(2):125-33.

S230. Steffen R, Reinhartz O, Blumhardt G, Bechstein WO, Raakow R, Langrehr JM, Rossaint R, Slama K, Neuhaus P. Bacterial and fungal colonization and infections using oral selective bowel decontamination in orthotopic liver transplantations. *Transpl Inter.* 1994;7(2):101-8.

S231. Stoutenbeek CP, Van Saene HK, Miranda DR, Zandstra DF. The effect of selective decontamination of the digestive tract on colonisation and infection rate in multiple trauma patients. Intens Care Med. 1984;10(4):185-92.

S232. Stoutenbeek CP, van Saene HK, Miranda DR, Zandstra DF, Langrehr D; The effect of oropharyngeal decontamination using topical nonabsorbable antibiotics on the incidence of nosocomial respiratory tract infections in multiple trauma patients. J Trauma 1987;27:357-364

S233. Vallés J, Peredo R, Burgueño MJ, de Freitas AP, Millán S, Espasa M, Martín-Loeches I, Ferrer R, Suarez D, Artigas A. Efficacy of single-dose antibiotic against early-onset pneumonia in comatose patients who are ventilated. *Chest*. 2013;143(5):1219-25.

S234. Veelo DP, Bulut T, Dongelmans DA, et al. The incidence and microbial spectrum of ventilator-associated pneumonia after tracheotomy in a selective decontamination of the digestive tract-setting. *J Infect* 2008; 56:20-6.

S235. Winter R, Humphreys H, Pick A, MacGowan AP, Willatts SM, Speller DC: A controlled trial of selective decontamination of the digestive tract in intensive care and its effect on nosocomial infection. J Antimicrob Chemother. 1992;30:73-87

S236. Abele-Horn M, Dauber A, Bauernfeind A, Russwurm W, Seyfarth-Metzger I, Gleich P, Ruckdeschel G: Decrease in nosocomial pneumonia in ventilated patients by selective oropharyngeal decontamination (SOD). Intensive Care Med. 1997;23:187-95.

S237. Acquarolo A, Urli T, Perone G, Giannotti C, Candiani A, Latronico N. Antibiotic prophylaxis of early onset pneumonia in critically ill comatose patients. A randomized study. Intensive Care Med. 2005; 31(4):510-6.

S238. Aerdts SJ, van Dalen R, Clasener HA, Festen J, van Lier HJ, Vollaard EJ: Antibiotic prophylaxis of respiratory tract infection in mechanically ventilated patients. A prospective, blinded, randomized trial of the effect of a novel regimen. Chest. 1991;100:783-791

S239. Bion JF, Badger I, Crosby HA, Hutchings P, Kong KL, Baker J, Hutton P, McMaster P, Buckels JA, Elliott TSJ: Selective decontamination of the digestive tract reduces gram-negative pulmonary colonization but not systemic endotoxemia in patients undergoing elective liver transplantation. Crit Care Med. 1994;22:40-49

S240. Blair P, Rowlands BJ, Lowry K, Webb H, Armstrong P, Smilie J Selective decontamination of the digestive tract: a stratified, randomized, prospective study in a mixed intensive care unit. Surgery 1991;110:303-309

S241. Blaise M, Pateron D, Trinchet JC, Levacher S, Beaugrand M, Pourriat JL. Systemic antibiotic therapy prevents bacterial infection in cirrhotic patients with gastrointestinal hemorrhage. Hepatology. 1994;20(1):34-8.

S242. Bouza E, Granda MJ, Hortal J, Barrio JM, Cercenado E, Muñoz P. Pre-emptive broad-spectrum treatment for ventilator-associated pneumonia in high-risk patients. *Intensive Care Med* 2013;39(9):1547-55.

S243. Camus C, Bellissant E, Sebille V, Perrotin D, Garo B, Legras A, Renault A, Le Corre P, Donnio PY, Gacouin A: Prevention of acquired infections in intubated patients with the combination of two decontamination regimens. *Crit Care Med* 2005, 33:307-314.

S244. Cerra FB, Maddaus MA, Dunn DL, Wells CL, Konstantinides NN, Lehmann SL, Mann HJ. Selective gut decontamination reduces nosocomial infections and length of stay but not mortality or organ failure in surgical intensive care unit patients. Arch Surg 1992;127:163-167.

S245. Cockerill FR, 3rd, Muller SR, Anhalt JP, et al. Prevention of infection in critically ill patients by selective decontamination of the digestive tract. *Ann Intern Med* 1992;117:545-53.

S246. de La Cal MA, Cerdá E, Garcıa-Hierro P, Van Saene HK. G ómez-Santos D, Negro E & Lorente JA. Survival benefit in critically ill burned patients receiving selective decontamination of the digestive tract: a randomized, placebocontrolled, double-blind trial. Ann Surg. 2005;241:424-30.

S247. Ferrer M, Torres A, Gonzalez J, Puig de la Bellacasa J, el-Ebiary M, Roca M, Gatell JM, Rodriguez-Roisin R: Utility of selective digestive decontamination in mechanically ventilated patients. Ann Intern Med. 1994;120:389-395

S248. Flaherty J, Nathan C, Kabins SA, Weinstein RA. Pilot trial of selective decontamination for prevention of bacterial infection in an intensive care unit. J Infect Dis 1990;162:1393-1397.

S249. Garbino J, Pichard C, Pichna P, Pittet D, Lew D, Romand J (2004) Impact of enteral versus parenteral nutrition on the incidence of fungal infections: a retrospective study in ICU patients on mechanical ventilation with selective digestive decontamination. Clinical Nutrition 2004;23;705-710.

S250. Gaussorgues P, Salord M, Sirodot S, Tigaud S, Cagnin S, Gerard M, Robert D. Efficiency of selective decontamination of the digestive tract on the occurrence of nosocomial bacteremia in patients on mechanical ventilation receiving betamimetic therapy. Réan Soins Intens Méd Urg 1991;7:169-174.

S251. Georges B, Mazerolles M, Decun J-F, Rouge P, Pomies S, Cougot P Décontamination digestive sélective résultats d'une étude chez le polytraumatisé. *Réanimation Soins Intensifs Médecin d'Urgence* 1994;3:621-627

S252. Hammond JM, Potgieter PD, Saunders LG. Selective decontamination of the digestive tract in multiple trauma patients-Is there a role? Results of a prospective, double-blind, randomized trial. Crit Care Med. 1994;22(1):33-9.

S253. Hellinger WC, Yao JD, Alvarez S, Blair JE, Cawley JJ, Paya CV, O'Brien PC. A randomized, prospective, double-blinded evaluation of selective bowel decontamination in liver transplantation. Transplantation 2002;73:1904-9.

S254. Jacobs S, Foweraker JE, Roberts SE: Effectiveness of selective decontamination of the digestive tract (SDD) in an ICU with a policy encouraging a low gastric pH. Clin Intensive Med. 1992;3:52-58

S255. Karvouniaris M, Makris D, Zygoulis P, Triantaris A, Xitsas S, Mantzarlis K, Petinaki E, Zakynthinos E. Nebulised colistin for ventilator-associated pneumonia prevention. Eur Resp J. 2015:46:1544-1547.

S256. Kerver AJ, Rommes JH, Mevissen-Verhage EA, et al Prevention of colonization and infection in critically ill patients: a prospective randomized study. Crit Care Med 1988;16:1087-1093.

S257. Korinek AM, Laisne MJ, Nicolas MH, Raskine L, Deroin V, Sanson-lepors MJ: Selective decontamination of the digestive tract in neurosurgical intensive care unit patients: a double-blind, randomized, placebo-controlled study. Crit Care Med. 1993;21:1466-73.

S258. Laggner AN, Tryba M, Georgopoulos A, Lenz K, Grimm G, Graninger W, Schneeweiss B, Druml W: Oropharyngeal decontamination with gentamicin for long-term ventilated patients on stress ulcer prophylaxis with sucralfate? *Wien Klin Wochenschr* 1994, 106:15-19.

S259. Langlois-Karaga A, Bues-Charbit M, Davignon A, Albanese J, Durbec O, Martin C, Morati N, Balansard G. Selective digestive decontamination in multiple trauma patients: cost and efficacy. *Pharmacy World and Science*. 1995;17(1):12-6.

S260. Palomar M, Alvarez-Lerma F, Jorda R, Bermejo B, Catalan Study Group of Nosocomial Pneumonia Prevention: Prevention of nosocomial infection in mechanically ventilated patients: selective digestive decontamination versus sucralfate. Clin Intens Care. 1997;8:228-235

S261. Quinio B, Albanese J, Bues-Charbit M, Viviand X, Martin C; Selective decontamination of the digestive tract in multiple trauma patients. A prospective double-blind, randomized, placebo-controlled study. Chest 1996;109:765-772

S262. Rimola A, Bory F, Teres J, Perez-Ayuso RM, Arroyo V, Rodes J. Oral, nonabsorbable antibiotics prevent infection in cirrhotics with gastrointestinal hemorrhage. *Hepatol*. 1985;5(3):463-7.

S263. Rocha LA, Martin MJ, Pita S, Paz J, Seco C, Margusino L, Villanueva R, Duran MT: Prevention of nosocomial infection in critically ill patients by selective decontamination of the digestive tract. A randomized, double blind, placebo-controlled study. Intensive Care Med. 1992;18:398-404

S264. Rodríguez-Roldán JM, Altuna-Cuesta A, López A, Carrillo A, Garcia J, León J, Martínez-Pellús AJ: Prevention of nosocomial lung infection in ventilated patients: use of an antimicrobial pharyngeal nonabsorbable paste. Crit Care Med. 1990;18:1239-42

S265. Rolando N, Gimson A, Wade J, Philpott-Howard J, Casewell M, Williams R: Prospective controlled trial of selective parenteral and enteral antimicrobial regimen in fulminant liver failure. Hepatol. 1993;17:196-201

S266. Rolando N, Wade JJ, Stangou A, Gimson AE, Wendon J, Philpott-Howard J, Williams R. Prospective study comparing the efficacy of prophylactic parenteral antimicrobials, with or without enteral decontamination, in patients with acute liver failure. Liver transplantation and surgery. 1996; 2:8-13

S267. Sanchez-Garcia M, Cambronero JA, Lopez-Diaz J, et al. Effectiveness and cost of selective decontamination of the digestive tract in critically ill intubated patients. A randomized, double-blind, placebo-controlled multicenter trial. *Am Rev Respir Dis* 1998; 158:908-16.

S268. Sirvent JM, Torres A, El-Ebiary M, Castro P, de Batlle J, Bonet A. Protective effect of intravenously administered cefuroxime against nosocomial pneumonia in patients with structural coma. *Am J Respir Crit Care Med* 1997;155:1729-1734

S269. Smith SD, Jackson RJ, Hannakan CJ, Wadowsky RM, Tzakis AG, Rowe MI; Selective decontamination in pediatric liver transplants. Transplantation 1993;55:1306-1308

S270. Stoutenbeek CP, van Saene HKF, Little RA, Whitehead A: The effect of selective decontamination of the digestive tract on mortality in multiple trauma patients: a multicenter randomized controlled trial. Intensive Care Med. 2007;33:261-270

S271. Ulrich C, Harinck-deWeerd JE, Bakker NC, et al Selective decontamination of the digestive tract with norfloxacin in the prevention of ICU-acquired infections: A prospective randomized study. Intensive Care Med 1989;15:424-431.

S272. Unertl K, Ruckdeschel G, Selbmann HK, et al; Prevention of colonization and respiratory infections in long-term ventilated patients by local antimicrobial prophylaxis. Intensive Care Med 1987;13:106-113

S273. Verwaest C, Verhaegen J, Ferdinande P, Schetz M, Van den Berghe G, Verbist L, Lauwers P: Randomized, controlled trial of selective digestive decontamination in 600 mechanically ventilated patients in a multidisciplinary intensive care unit. Crit Care Med. 1997;25:63-71

S274. Wiener J, Itokazu G, Nathan C, Kabins SA, Weinstein RA: A randomized, double-blind, placebo-controlled trial of selective digestive decontamination in a medical-surgical intensive care unit. Clin Infect Dis. 1995;20:861-867

S275. Ables AZ, Blumer NA, Valainis GT, Godenick MT, Kajdasz DK, Palesch YY. Fluconazole Prophylaxis of Severe Candida Infection in Trauma and Postsurgical Patients: A Prospective, Double-Blind, Randomized, Placebo-Controlled Trial. Infect Dis Clin Practice 2000;9(4):169-175.

S276. Eggimann P, Francioli P, Bille J, Schneider R, Wu MM, Chapuis G, Chiolero R, Pannatier A, Schilling J, Geroulanos S, et al (1999) Fluconazole prophylaxis prevents intra-abdominal candidiasis in high-risk surgical patients. Crit Care Med 27:1066–1072.

S277. Giglio M, Caggiano G, Dalfino L, Brienza N, Alicino I, Sgobio A, Favale A, Puntillo F. Oral nystatin prophylaxis in surgical/trauma ICU patients: a randomised clinical trial. Crit Care 2012;16(2):R57.

S278. Jacobs S, Price Evans DA, Tariq M, Al Omar NF (2003) Fluconazole improves survival in septic shock: a randomized double-blind prospective study. Crit Care Med 31:1938–1946.

S279. Lumbreras C, Cuervas-Mons V, Jara P, Del Palacio A, Turrion VS, Barrios C, Paya C V (1996) Randomized trial of fluconazole versus nystatin for the prophylaxis of *Candida* infection following liver transplantation. J Infect Dis 174(3); 583-588.

S280. Normand S, François B, Dardé ML, Bouteille B, Bonnivard M, Preux PM, Gastinne H, Vignon P. Oral nystatin prophylaxis of *Candida* spp. colonization in ventilated critically ill patients. Intensive Care Med 2005;31:1508–1513.

S281. Ostrosky-Zeichner L, Shoham S, Vazquez J, Reboli A, Betts R, Barron MA, Pappas P G. MSG-01: a randomized, double-blind, placebo-controlled trial of caspofungin prophylaxis followed by pre-emptive therapy for invasive candidiasis in high-risk adults in the critical care setting. Clin Infect Dis 2014;58(9):1219-1226.

S282. Savino JA, Agarwal N, Wry P, Policastro A, Cerabona T, Austria L. Routine prophylactic antifungal agents (clotrimazole, ketoconazole, and nystatin) in nontransplant nonburned critically ill surgical and trauma patients. J Trauma 1994;36:20–26.

S283. Schuster MG, Edwards JE, Sobel JD, Darouiche RO, Karchmer AW, Hadley S, Rex JH (2008) Empirical fluconazole versus placebo for intensive care unit patients: a randomized trial. Ann Intern Med 149(2):83-90.

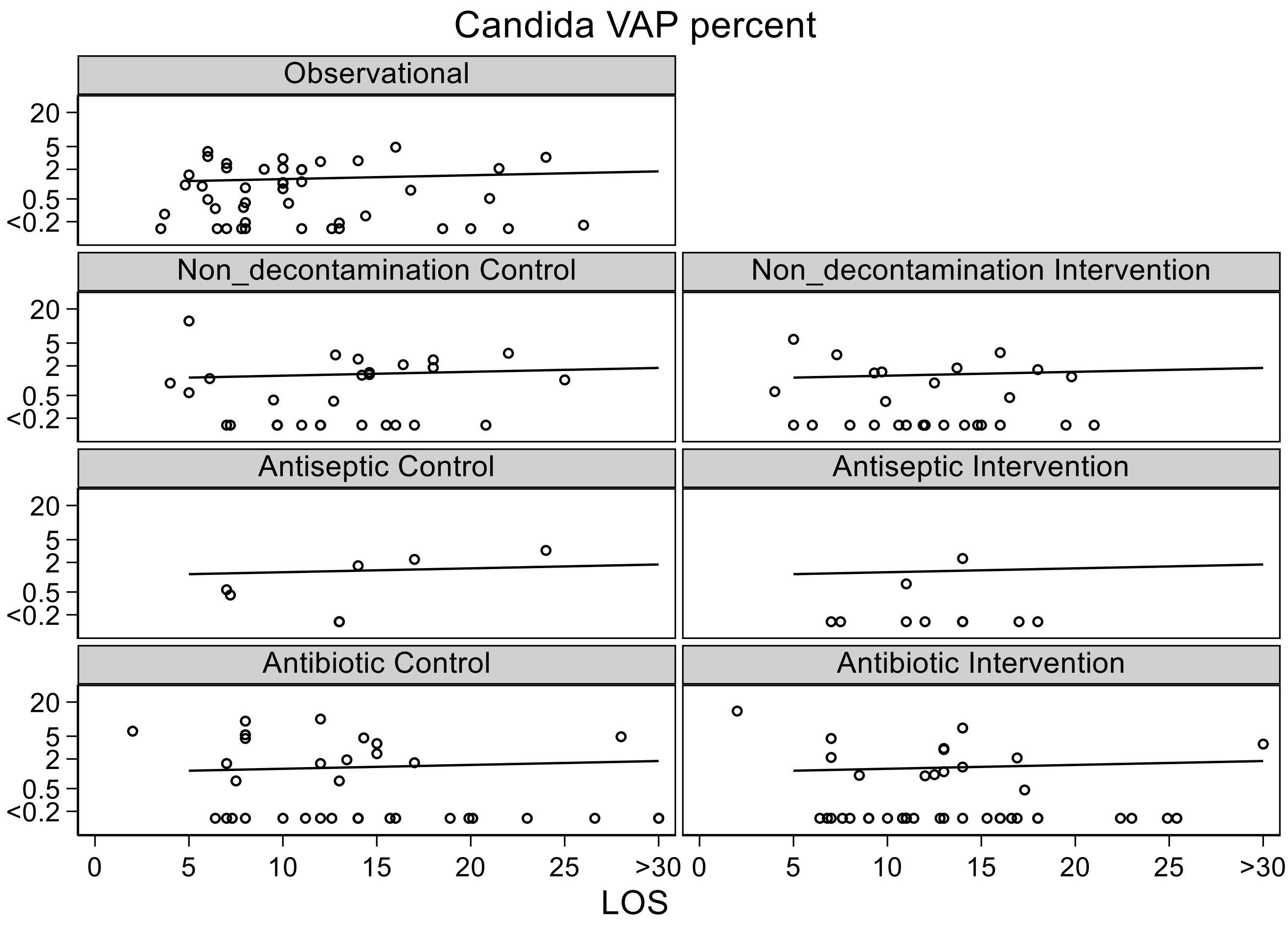

Figure S1 Candida RT count data versus group mean length of stay (LOS; days) in component (control and intervention) groups of various methods of infection prevention in the ICU. In each panel, the linear regression line derived from the observation studies is shown as a benchmark. The panels for the SAF studies are not shown as there were fewer than 3 observations in each.

Fig S2 GSEM model 1 The three-part boxes represent the count data for Candida, and S aureus, CNS and Enterococci as VAP (v_can_n, v_S aureus_n) and BSI as bacteremia (b_S aureus_n, b_cns_n, b_Ent_n) of candidemia (b_can_n) isolates. The circles contain error terms (ε). (PPAP is protocolized parenteral antibiotic prophylaxis; a_S is topical antiseptic prophylaxis, TAP is topical antibiotic prophylaxis; non-D is a non-decontamination intervention; Crf = Candidemia risk factor; Trauma ICU arbitrarily defined as an ICU for which >50% of admissions were for trauma; LOS is length of ICU stay; MVP90 is use of mechanical ventilation by more than 90% of the group)

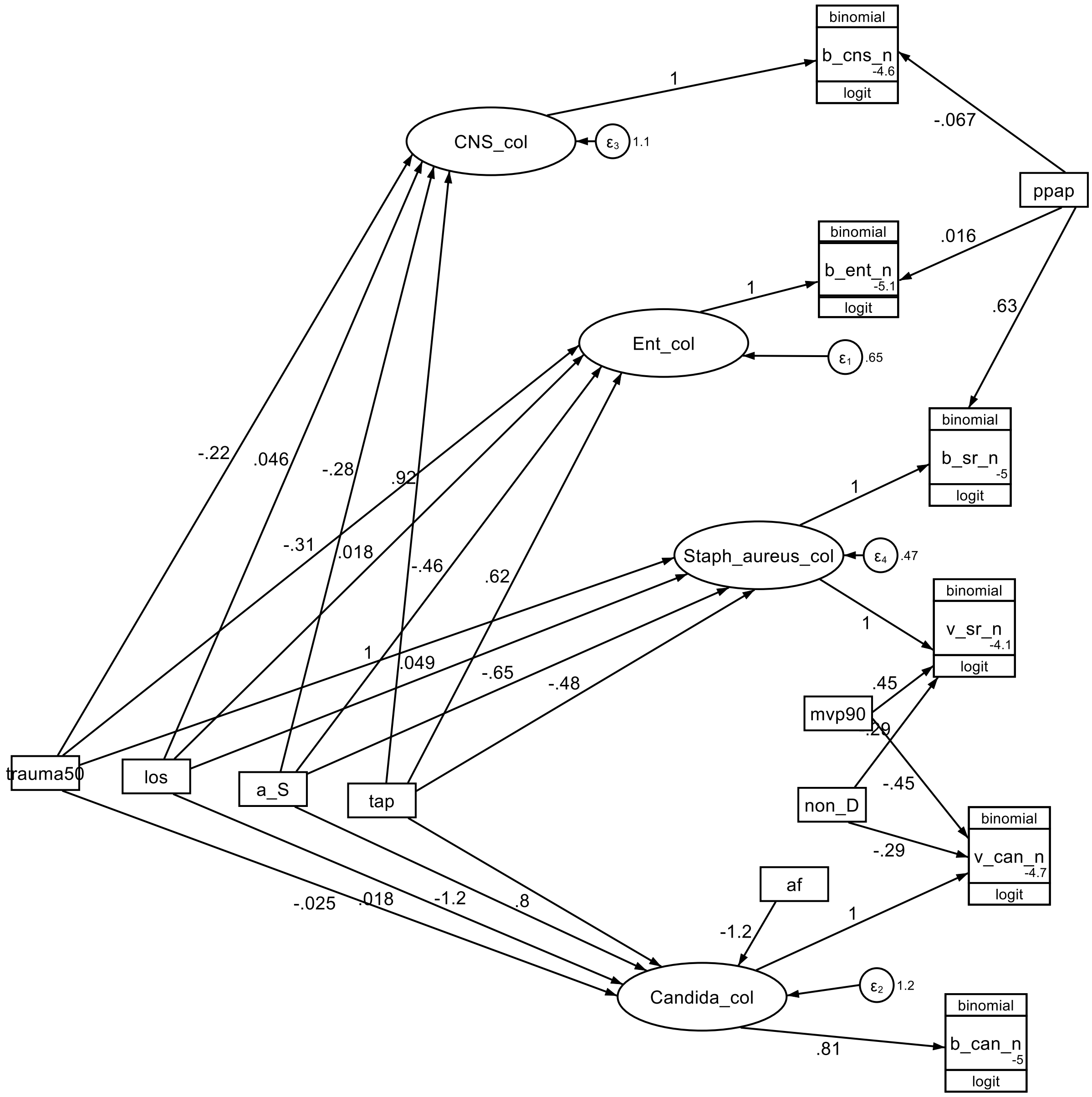

Fig S3 GSEM model 2 *(see Fig S2 for abbreviations)*

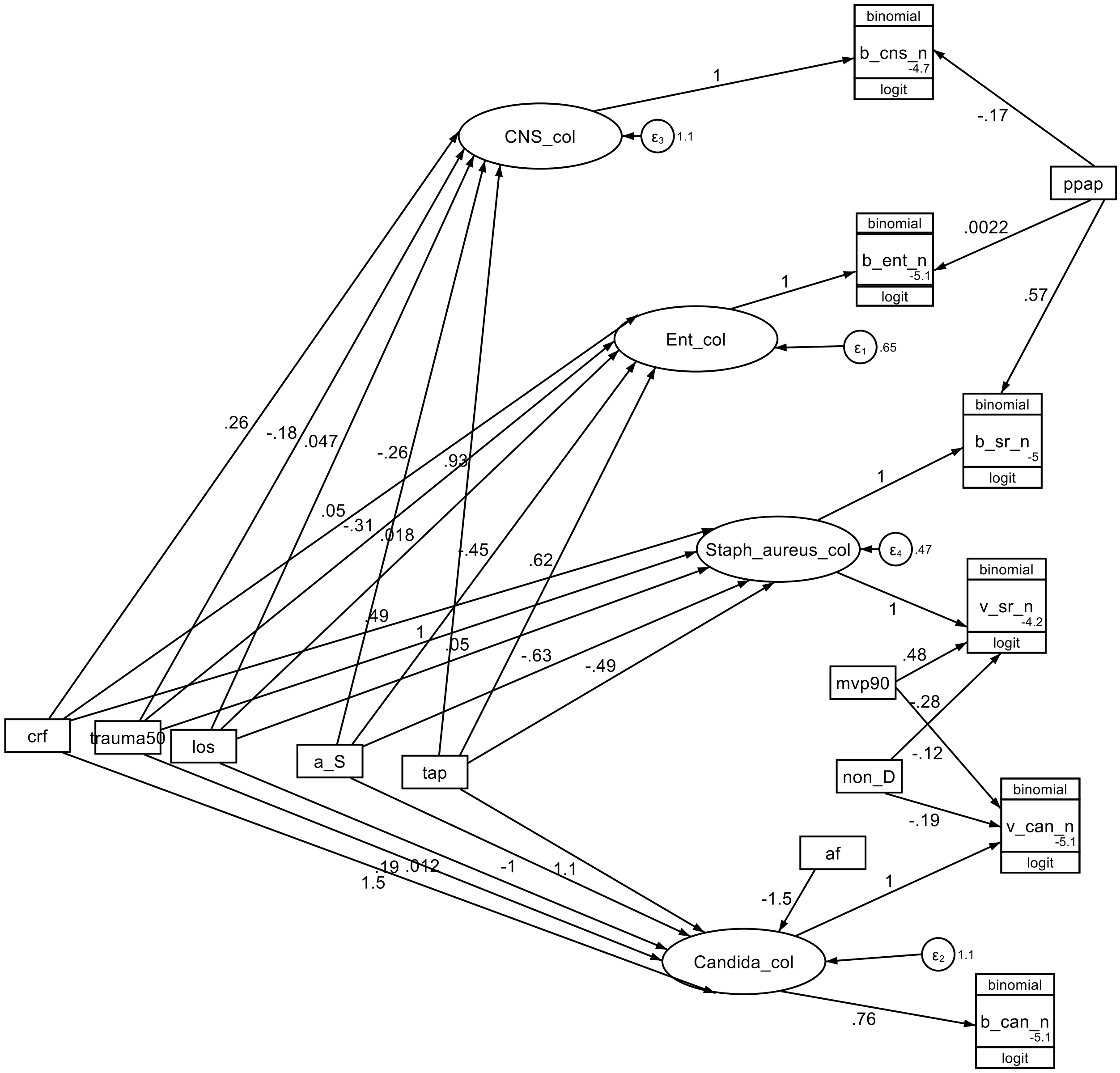

Fig S4 GSEM model 3 *(see see* Fig *S2 for abbreviations)*

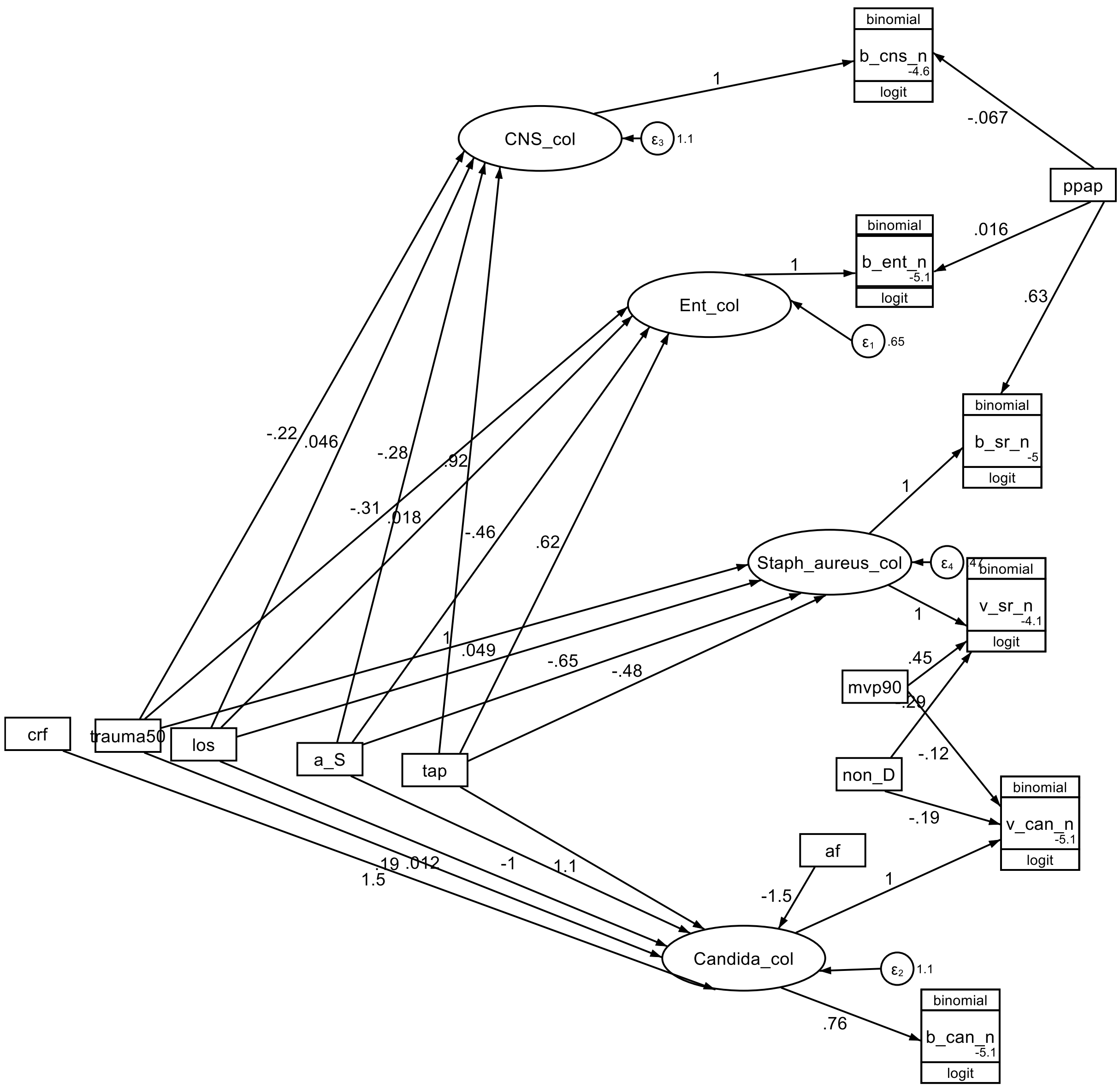

Supplement: Supplementary file 1 — Additional file 1: Table S1. Observational studies (Benchmark groups). Table S2. Groups of non-decontamination studies. Table S3. Groups of Anti-septic studies. Table S4. Groups of Antibiotic (= TAP ± PPAP) studies. Table S5. Groups from single anti-fungal (SAF) studies. Reference list for included studies [Ref S1 - S283]. Fig S1. Candida RT count data. Fig S2. GSEM model. Fig S3. GSEM model. Fig S4. GSEM model. [file 12982_2022_116_MOESM1_ESM.pdf]
